# Targeting PRMT5 Inhibitor–Induced Adaptation in Pancreatic Cancer with the RBM39 Degrader Indisulam

**DOI:** 10.1158/2767-9764.CRC-25-0670

**Published:** 2026-09-01

**Authors:** Valentina Spielmann, Jonas Buchloh, Selen Selcen, Carolin Schneider, Shaishavi Jansari, Xin Fang, Ningjun Duan, Engin Demirdizen, Lukas Krauß, Jessica Eggert, Geraldine Siegfried, Sandrine Fedou, Christof Lenz, Lena Wieland, Lena-Christin Conradi, Maximilian Reichert, Volker Ellenrieder, Michael Ghadimi, Marian Grade, Elisabeth Hessmann, Abdel-Majid Khatib, Christian J. Braun, Florian Wegwitz, Dieter Saur, Matthias Wirth, Günter Schneider

**Affiliations:** 1 Department of General, Visceral and Pediatric Surgery, University Medical Center Göttingen, Göttingen, Germany.; 2 Department of Gynecology and Obstetrics, University Medical Center Göttingen, Göttingen, Germany.; 3 Bordeaux Institute of Oncology (BRIC)-UMR1312, University of Bordeaux, Bordeaux, France.; 4 XenoFish, University of Bordeaux, Bordeaux, France.; 5 Department of Clinical Chemistry, University Medical Center Göttingen, Göttingen, Germany.; 6 Bioanalytical Mass Spectrometry Group, Max Planck Institute for Multidisciplinary Sciences, Göttingen, Germany.; 7 Clinical Research Unit 5002, KFO5002, University Medical Center Göttingen, Göttingen, Germany.; 8 CCC-N (Comprehensive Cancer Center Lower Saxony), Göttingen, Germany.; 9 Medical Clinic and Polyclinic II, Klinikum rechts der Isar, Technical University Munich, Munich, Germany.; 10 Translational Pancreatic Research Cancer Center, Medical Clinic and Polyclinic II, Klinikum Rechts Der Isar, Technical University Munich, Munich, Germany.; 11 Center for Protein Assemblies (CPA), Technical University of Munich, Garching, Germany.; 12 Center for Organoid Systems and Tissue Engineering (COS), Technical University Munich, Garching, Germany.; 13 German Cancer Consortium (DKTK), partner site Munich, a partnership between DKFZ and University Hospital Klinikum rechts der Isar, Munich, Germany.; 14 DEFEAT-PDAC – Decoding and Targeting the PDAC Ecosystem – a German Pancreatic Cancer Alliance (GPCA) Consortium – partner site Munich, Munich, Germany.; 15 Department of Gastroenterology, Gastrointestinal Oncology and Endocrinology, University Medical Center Göttingen, Göttingen, Germany.; 16 Department of Pediatrics, Dr. von Hauner Children’s Hospital, University Hospital, LMU Munich, Munich, Germany.; 17 Department of Pediatrics, Goethe University Frankfurt, Frankfurt, Germany.; 18 Institute for Translational Cancer Research and Experimental Cancer Therapy, Technical University Munich, Munich, Germany.; 19 Department of Hematology, Oncology and Cancer Immunology, Campus Benjamin Franklin, Charité - Universitätsmedizin Berlin, Corporate Member of Freie Universität Berlin and Humboldt-Universität zu Berlin, Berlin, Germany.; 20 Max-Delbrück-Center for Molecular Medicine, Berlin, Germany.; 21 German Cancer Consortium (DKTK), partner site Berlin, a partnership between DKFZ and Charité - Universitätsmedizin Berlin, Berlin, Germany.; 22 DEFEAT-PDAC – Decoding and Targeting the PDAC Ecosystem – a German Pancreatic Cancer Alliance (GPCA) Consortium – partner site Göttingen, Göttingen, Germany.

## Abstract

Pancreatic ductal adenocarcinoma (PDAC) remains a formidable clinical challenge. Next-generation protein arginine methyltransferase 5 (PRMT5) inhibitors show promising clinical results in a subset of PDACs with codeletion of the tumor-suppressor *CDKN2A* and the methylthioadenosine phosphorylase (*MTAP*) gene, but resistance limits their efficacy. Our study suggests that compensatory spliceosomal reprogramming contributes to adaptation to PRMT5 inhibition. Through comprehensive molecular profiling, we demonstrate that PRMT5 inhibitors induce upregulation of RNA-binding proteins, including RNA-binding protein 39 (RBM39). We investigated whether this response could be therapeutically leveraged by combining PRMT5 inhibition with indisulam-mediated RBM39 degradation, which yielded synergistic activity in cellular model systems. The combination strategy significantly enhanced apoptotic cell death and suppressed tumor outgrowth in resistance assays compared with single-agent treatments. Multiomics analysis revealed concomitant suppression of DNA repair and metabolic pathways. Collectively, our work support spliceosomal rewiring as a candidate adaptive response to PRMT5 inhibition and nominates RBM39 as a candidate therapeutic vulnerability, thereby supporting further evaluation of dual targeting of the splicing machinery.

**Significance:**

Our study suggests that compensatory spliceosomal reprogramming occurs in response to PRMT5 inhibition. We investigated this vulnerability by combining PRMT5 inhibition with indisulam-mediated RBM39 degradation, which yielded synergistic antitumor activity in selected cellular PDAC models.

## Introduction

The protein arginine methyltransferase (PRMT) family consists of nine members, which utilize the methyl donor S-adenosyl methionine (SAM) to catalyze arginine monomethylation (MMA), asymmetric dimethylation, and symmetric dimethylation (sDMA; ref. 1). The main type II enzyme, PRMT5, catalyzes the formation of MMA and sDMA and functions with its cofactor, methylosome protein 50 (MEP50), to regulate a variety of important cellular functions, including RNA splicing, epigenetic control and transcription, DNA repair, and translation (1). In pancreatic ductal adenocarcinoma (PDAC), PRMT5 is frequently overexpressed and associated with aggressive tumor behavior, poor survival, and therapy resistance (2). Preclinical studies highlight PRMT5 inhibition as a promising strategy in PDAC (3–8). However, first-generation PRMT5 inhibitors (PRMT5i) like JNJ-64619178 and GSK-3326595 demonstrated significant dose-limiting toxicities, particularly hematologic adverse effects, while showing only modest clinical activity in early trials (2). In 2016, a synthetic lethal interaction was discovered between methylthioadenosine phosphorylase (*MTAP*) deletion and PRMT5 (9–11). MTAP, a key enzyme in the methionine salvage pathway, is frequently codeleted with the tumor-suppressor *CDKN2A* in cancers, including 20% to 25% of PDAC cases. This deletion results in the accumulation of methylthioadenosine (MTA), a natural inhibitor of PRMT5 (9–11). Consequently, *MTAP*-deficient cancer cells develop a heightened dependency on PRMT5 (9–11). These insights led to the development of MTA-cooperative PRMT5is (e.g., MRTX1719, which was recently renamed as BMS-986504 (12, 13), AMG193 (14), or TNG462 (15), which offer an extended therapeutic window and selective targeting of *MTAP*-null tumors (2). Although MTA-cooperative PRMT5is have demonstrated clinical activity in PDAC, including partial responses, their objective response rates of ∼20% across all cancers with *MTAP* deletion (16, 17) and eventual tumor progression highlight the necessity for rational combination strategies to improve therapeutic efficacy.

Acquired resistance remains a major limitation of targeted cancer therapies and commonly arises through complex, interconnected processes (18). In addition to genetic mechanisms and clonal selection processes, emerging evidence indicates that cancer cells can survive therapeutic pressure through adaptive transcriptional reprogramming, thereby maintaining fitness under drug exposure. Importantly, recent studies have described an adaptive “resistance continuum,” in which drug-induced cellular states not only promote survival but also create new, targetable dependencies (19). The cellular adaptation processes induced by PRMT5i treatment that limit the efficacy of these compounds remain poorly understood. However, given the encouraging early clinical data for MTA-cooperative PRMT5is (16, 17, 20, 21), there is an unmet need to elucidate the mechanisms underlying primary, adaptive, and acquired resistance. We therefore sought to determine whether adaptive mechanisms occur in response to PRMT5 inhibition in PDAC. Here, we show that PRMT5 blockade is associated with upregulation of components of the splicing machinery, suggesting a potentially targetable vulnerability.

## Materials and Methods

### Drugs

PRMT5is GSK-3326595 (#HY-101563), JNJ-64619178 (#HY-101564), and MRTX1719 (#HY-139611) were purchased from MedChemExpress. Indisulam was purchased from Selleck Chemicals (#S9742). Vopimetostat/TNG462 was purchased from HyCultec (#HY-156680). Dimethyl sulfoxide (DMSO) was purchased from Merck (#1.02931, Merck KGaA).

### Cell culture, authentication, and *Mycoplasma* testing

Human pancreatic cell lines were cultured at 37°C, 5% CO_2_ according to their specific requirements. Panc-1 (RRID: CVCL_0480), MiaPaCa-2 (RRID: CVCL_0428), PaTu-8988T (RRID: CVCL_1847), and MYC CRISPRa human pancreatic adenocarcinoma (HPAC) cells derived from HPAC cells (RRID: CVCL_3517) were cultivated in Dulbecco’s modified Eagle medium (DMEM) high-glucose (#D5796, Sigma-Aldrich) with 10% (v/v) fetal bovine serum (FBS; #F7524, Merck KGaA) as recently described (4). PSN1 (RRID: CVCL_1644), DanG (RRID: CVCL_0243), and BxPc3 (RRID: CVCL_0186) cells were cultivated in RPMI-1640 medium (#D8758, Sigma-Aldrich) and supplemented with 10% (v/v) FBS. HPAC (RRID: CVCL_3517) cells were cultured in DMEM/Nutrient Mixture F12 Ham (#D8437, Sigma-Aldrich) with 5% (v/v) FBS. Cells were kept to a maximum of 15 passages between thawing and usage for experiments. Conventional PDAC cell lines were authenticated by Multiplexion (Multiplexion GmbH) using the multiplex human cell line authentication test at various time points, depending on the cell line (2019, 2022, 2023, or 2025). Cell lines were obtained and transferred from the Medical Clinic and Polyclinic II, Technical University of Munich. Panc-1 and PSN1 cells containing the split green fluorescence protein (GFP)-based fluorogenic caspase reporter system (22) were recently described (23).

Patient-derived cell lines (PDCL) were generated from patient-derived xenotransplant PDAC models (24) in the framework of the CRU5002 consortium (https://gccc.umg.eu/en/cru-5002/) and, for the experiments described here, cultured in RPMI-1640 (#D8758, Sigma-Aldrich) containing 10% (v/v) FCS. All cell lines were routinely screened for *Mycoplasma* contamination using a PCR-based protocol, as previously described (25).

### Clonogenic growth assay

For clonogenic growth assays, 1,000 to 20,000 PDAC cells were seeded in 24-well plates (#83.3922.005, Sarstedt AG & Co. KG) and treated with various therapies 1 day later. Cells were incubated until vehicle (DMSO)-treated controls reached confluence or as indicated. Subsequently, the medium was aspirated, and cells were washed once with phosphate buffered saline (PBS; #12579099, Fisher Scientific GmbH). For staining, 200 μL of Crystal Violet solution [0.2% w/v Crystal Violet (#T123.3, Carl Roth GmbH + Co. KG) and 2% ethanol (#9065.2, Carl Roth GmbH + Co. KG)] were added to each well and incubated for 10 to 15 minutes at room temperature (RT) on a platform shaker. Wells were then washed twice with 800 μL of ddH_2_O, and plates were air-dried before scanning at 600 dpi. To quantify clonogenic growth, the crystal violet dye was solubilized in 600 μL of 1% (w/v) sodium dodecyl sulfate (SDS) solution (#CN30.3, Carl Roth GmbH + Co. KG). Absorbance was measured at 570 nm using a Multilabel Plate Reader (Victor X4, PerkinElmer). Growth inhibition was normalized to the DMSO control for analysis.

### *In situ* resistance assay

The *in situ* resistance assay (ISRA) was performed as previously described (26, 27). Before starting, the (growth-inhibitory concentration) GI_80_ values of the monotherapies as well as the combination therapies were determined. Briefly, 25 PSN1 cells were seeded per well in a transparent 96-well plate with 100 μL of the respective medium. After 24 hours, cells were treated with the GI_80_ value of the indicated drugs or the GI_80_ value for the combination therapy (30 wells per condition). Starting on day 7, confluency was assessed weekly using the Celigo Image Cytometer (Revvity Germany Diagnostics GmbH). The medium containing treatments was changed weekly. Drug treatment and confluency measurements were repeated over 10 weeks, with resistance defined as a well reaching ≥50% confluency. Data were used to plot Kaplan–Meier curves using GraphPad Prism v10, and significance was determined with a log-rank test. From each monotherapy and vehicle control coculture, three populations (after they reached ≥50% confluency) were detached and expanded for further analysis. These populations were cultivated in medium containing the drug and seeded for clonogenic growth experiments and for protein isolation.

### siRNA transfection

The cells were transfected with a control esiRNA and esiRBM39 as described (28). Negative control (FLUC, #EHUFLUC) and RNA-binding protein 39 (RBM39; #EHU070351) esiRNAs were purchased from Eupheria Biotech (Eupheria Biotech GmbH).

### Western blot

The culture medium was removed, and cells were washed once with ice-cold PBS. Cells were lysed using M-PER Mammalian Protein Extraction Reagent (#78502, Thermo Fisher Scientific) supplemented with protease inhibitor (#04693116001, Merck KGaA) and phosphatase inhibitor (#04906837001, Merck KGaA). Protein concentration was determined using Bradford assay (#2924, Carl Roth GmbH + Co. KG), and equal amounts of protein were mixed with Laemmli buffer [8% (v/v) glycerol (#15514011, Thermo Fisher Scientific), 0.8% (w/v) SDS (#CN30.3, Carl Roth GmbH + Co. KG), 0.01% (w/v) bromophenol blue (#B0126, Merck KGaA), and 1% (v/v) 2-mercaptoethanol (#M6250, Sigma-Aldrich) in 250 mmol/L TRIS (#4855.3, Carl Roth GmbH + Co. KG), pH 8.6]. Samples were boiled at 95°C for 5 minutes and stored at −20°C. Proteins were separated on SDS–polyacrylamide gels using the Mini-PROTEAN Tetra system (Bio-Rad Laboratories). Prestained protein ladders (#26617 or #26619, Thermo Fisher Scientific) were included as a molecular weight reference. Proteins were transferred to polyvinylidene difluoride membranes (#GE10600023, Merck KGaA) via wet transfer. The membranes were blocked for 2 hours in 5% nonfat milk (#T145.4, Carl Roth GmbH + Co. KG) in TRIS-buffered saline [TBS; to 1 L in ddH_2_O: 6.057 g TRIS (#4855.3, Carl Roth GmbH + Co. KG) and 8.8 g NaCl (#3957.2 Carl Roth GmbH + Co. KG), pH 7.6] with 0.1% Tween 20 (#9127.2, Carl Roth GmbH + Co. KG; TBS-T) and then incubated overnight at 4°C with the following primary antibodies: β-actin (RRID: AB_310594, 1:5,000 in 5% milk, mouse, #A5316, Sigma-Aldrich), HSP90 (RRID: AB_675659, 1:2,000 in 5% milk, mouse, #Sc-113119, Santa Cruz Biotechnology), RBM39 (RRID: AB_1079749, 1:1,000 in 5% BSA, rabbit, #HPA001591, Sigma-Aldrich), PRMT5 (RRID: AB_2799945, 1:1,000 in 5% BSA, rabbit, #79998, Cell Signaling Technology), or MTAP (RRID: AB_1904054, 1:1,000 in 5% BSA, #4158, Cell Signaling Technology). After washing in TBS-T three times, membranes were incubated for 1 hour at RT with the following horseradish peroxidase (HRP)-conjugated secondary antibodies: anti-rabbit polyclonal antibody (RRID: AB_10262463, 1:30,000 in 5% milk, #R1364HRP, OriGene Technologies) or anti-mouse polyclonal antibody (RRID: AB_10266129, 1:30,000 in 5% milk, #R1253HRP, OriGene Technologies). Signal detection was performed using Immobilon Forte Western HRP substrate (#WBLUF0500, Merck KGaA), and chemiluminescence was detected using the ODYSSEY XF imaging system (LI-COR Biotech). Protein bands were quantified using Image J (US National Institutes of Health). Protein expression values were normalized on expression of a housekeeping gene (β-actin or HSP90), and the final expression values were calculated out of three biological replicates.

### Caspase-3/7 assay

Caspase-3/7 activity was measured using the Caspase-Glo 3/7 Assay (#G8093, Promega) following the manufacturer’s protocol. For normalization, cell viability was assessed in parallel using the CellTiter-Glo Luminescent Cell Viability Assay (#G7573, Promega). Luminescence was quantified using a Victor X4 Multilabel Plate Reader (PerkinElmer). The luminescence signal from each assay was normalized to the DMSO control (set as 100%). Caspase-3/7 activity was then further normalized to cell viability to account for differences in cell number.

### Cell-cycle analysis by flow cytometry

Cells were seeded 1 day prior to drug treatment and harvested 48 or 72 hours after treatment. Cells were detached using either 0.05% ethylenediaminetetraacetic acid (EDTA; #P10-026100, PAN-Biotech GmbH) or 0.25% Trypsin-EDTA (#25200-072, Thermo Fisher Scientific), resuspended in medium, and transferred to a 15-mL reaction tube (#62.554.502, Sarstadt AG & Co. KG). The cell suspension was centrifuged at 1,000 rpm (4°C, 5 minutes), and the pellet was resuspended in 500 μL cold PBS followed by the addition of 2 mL 70% ethanol and stored at 4°C for at least 1 hour for fixation. Fixed cells were washed twice with 2 mL FACS buffer [1% (v/v) FBS and 2 mmol/L EDTA in PBS] or PBS containing 0.05% Triton X-100 (#T8787 Merck KGaA), with centrifugation at 1,000 rpm (4°C, 5 minutes). Cells were then resuspended in 0.5 mL FACS buffer and treated with 2.5 μL RNase A (EN0531, Thermo Fisher Scientific) for 1 hour at 37°C. Following RNase treatment, 5 μL propidium iodide (PI; #556547, BD Biosciences) was added, and samples were incubated for 15 minutes in the dark. For each sample, 30,000 events were acquired using a CytoFLEX S flow cytometer (Beckman Coulter). Data analysis was performed using FlowJo v10.8 (RRID: SCR_008520; FlowJo LLC), where single cells were gated, and cell-cycle phases were determined based on PI signal intensity.

### H2A.X phosphorylation assay kit (flow cytometry)

A total of 200,000 cells were seeded 1 day prior to drug treatment. For positive control, 1 day after seeding, cells were irradiated with 2 Gy (XCELL225, Kubtec) for 30 minutes. Following 48 or 72 hours of treatment, the γH2A.X phosphorylation assay was performed using the H2A.X Phosphorylation Assay Kit (#17-344, Merck KGaA) according to the manufacturer’s protocol. In brief, cells were trypsinized and combined with their corresponding supernatant and then centrifuged at 800 rpm for 5 minutes at RT. The cell pellet was washed three times with PBS, and cells were resuspended in 500 μL of 1X Fixation Solution and incubated on ice for 20 minutes. Cells were then washed twice with 1X PBS, using a volume three times that of the original culture medium. The fixed cell pellet was gently resuspended in 50 μL of Permeabilization Solution and transferred to a 1.5-mL tube. Either 3.5 μL of anti-phospho-histone H2A.X (Ser139)-FITC conjugate or mouse IgG-FITC [negative control; both antibodies are included in the H2A.X phosphorylation Assay Kit (#17-344, Merck KGaA)] was added to the cell suspension. Samples were incubated on ice for 20 minutes with gentle agitation to ensure uniform staining followed by washing by adding 100 μL of 1X Wash Solution. Afterward, cells were centrifuged, and the supernatant was removed. For DNA counterstaining, cells were resuspended in 300 μL of FACS buffer (PBS + 1% FBS). PI (10 μg/mL, #P4864, Sigma-Aldrich) and RNase A 1 μg/mL (#19101, Qiagen) were added to each sample. The samples were then analyzed using a CytoFLEX S Flow Cytometer (Beckman Coulter), with 30,000 events acquired per sample. Single cells were gated, and the FITC signal was used to identify γH2A.X-positive cells. Data were analyzed using FlowJo v10.8 software (RRID: SCR_008520; FlowJo LLC).

### Live-cell imaging of caspase activity

Caspase-3/7 activity was monitored in real-time using the IncuCyte Zoom Live-Cell Analysis System (Sartorius AG) as described (23). In brief, Panc-1 and PSN1 caspase reporter cells (23), stably expressing a fluorescent caspase-3/7 reporter construct (22), were seeded in six technical replicates per condition in transparent, flat-bottom 96-well plates (Sarstedt) and allowed to adhere overnight (37°C, 5% CO_2_). Apoptosis was induced with 400 nmol/L indisulam, 8 nmol/L JNJ-64619178, or 100 nmol/L MRTX1719 with DMSO serving as the vehicle control. The system simultaneously tracked GFP (indicating caspase-3/7 activation) and orange fluorescence (mCherry; marking the total cell population). Images were acquired every 8 hours for the indicated time points. Each condition was tested in biological triplicates. Fluorescence intensity was quantified using the IncuCyte analysis software (Sartorius AG).

### RNA sequencing and gene set enrichment analysis

Cells were seeded 1 day prior to treatment with the indicated drugs. RNA was isolated using the RNeasy Mini Kit (#74106, QIAGEN), and the RNA was eluted with nuclease-free water and stored at −80°C. Sequencing was conducted by Novogene (Novogene GmbH). The sequencing quality was assessed using FastQC (http://www.bioinformatics.babraham.ac.uk/projects/fastqc/). Raw RNA-seq reads were then mapped to the human hg38 genome build using STAR aligner v.2.5.3a (RRID: SCR_004463; ref. 29) with the following parameters:

STAR –runMode alignReads –runThreadN 6 –genomeDir hg38 –twopassMode Basic –outSAMtype BAM SortedByCoordinate –outFilterMultimapNmax 20 –outFilterMismatchNmax 999 –outFilterMismatchNoverLmax 0.06 –alignIntronMin 70 –alignIntronMax 500000 –alignMatesGapMax 500000 –alignSJoverhangMin 8 –alignSJDBoverhangMin 1 –outSAMstrandField intronMotif –outFilterType BySJout –readFilesCommand gunzip -c.

Exonic reads were counted using the featurecount (RRID: SCR_012919; ref. 30) function of subread v.2.0.0 (RRID: SCR_009803; ref. 31) using GRCh38.112 annotations and parameters: featureCounts -T 12 -p -t exon -g gene_id.

Subsequently, differential gene expression analysis was performed in RStudio (R v4.1; RRID: SCR_000432) using DEseq2 v1.42.1 (32). Lowly expressed genes were filtered [sum (read counts) < *n*; *n* = number of samples], and read counts were normalized and transformed using variance-stabilizing transformation. For differential analysis, log_2_ fold change (FC) were shrunken to account for larger dispersion in lowly expressed genes using apeglm (33). Gene set enrichment analysis (GSEA) was performed with GeneTrail3.2 (RRID: SCR_006250; ref. 34) or the GSEA 4.4.0 Desktop App (RRID: SCR_003199; Broad Institute, Massachusetts Institute of Technology, www.gsea-msigdb.org/gsea/index.jsp; ref. 35).

The RNA sequencing (RNA-seq) data from our previous project (20 nmol/L JNJ-64619178–treated PSN1 and DanG cells for 72 hours, HPAC-MYC CRISPRa; ref. 4) can be accessed via ENA: PRJEB43040. Data of AM-9747–treated PDAC cell lines were accessed via NCBI Gene Expression Omnibus (GEO): GSE273376.

### Data repositories and public dataset analyses

Clinical Proteomic Tumor Analysis Consortium (CPTAC) proteomic data for PDAC [*n* = 140, CPTAC Pancreatic Ductal Adenocarcinoma Discovery Study (CPTAC_PDAC); ref. 36] were accessed via the LinkedOmics platform (37). The correlation between RBM39 and PRMT5 protein expression was analyzed in a dataset downloaded from LinkedOmics. Subsequently, GSEA was performed on their associated pathways using LinkInterpreter with Gene Ontology Biological Processes (GO-BP) signatures, using *P* value as the ranking criterion (parameters: minimum number of genes = 3, simulations = 1,000). Indisulam sensitivity (log_2_ FC PRISM repurposing public Q23) and DCAF15 mRNA expression (log_2_[Transcripts Per Million (TPM) + 1]) as well as mRNA expression data of human conventional PDAC lines (CCLE_expression; Q3/19) were downloaded from the DepMap (https://depmap.org/portal/) portal (RRID: SCR_017655; ref. 38). For the correlation of RBM39 and PRMT5 mRNA expression data from three PDAC datasets—PRINCE Trial 2022 (39), The Cancer Genome Atlas GDC 2025 (40), and CPTAC 2021 (36)—were accessed via cBioPortal (RRID: SCR_014555; refs. 41, 42).

### Alternative splicing analysis

To assess drug-induced alterations in mRNA maturation, alternative splicing analysis was performed using rMATS turbo (v4.3.0; ref. 43). Paired-end RNA-seq data (read length: 150 bp, ENA: PRJEB95000) from PSN1 cells treated for 48 hours with 4 nmol/L JNJ-64619178 and 200 nmol/L indisulam, either as single agents or in combination, as well as untreated controls, were analyzed using the parameters -t paired and –readLength 150. Differential alternative splicing events—including skipped exons, retained introns, alternative 3′ splice sites, alternative 5′ splice sites, and mutually exclusive exons— were identified. Significant splicing events were defined by a false discovery rate (FDR) ≤ 0.05 and an absolute change in percent spliced-in (|ΔPSI|) ≥ 0.1.

### Proteome analysis

Cells were seeded 1 day prior to treatment with either the combination therapy (4 nmol/L JNJ-64619178 + 200 nmol/L indisulam) or DMSO control for 96 hours. The cell pellet was obtained and frozen in liquid nitrogen and further stored at −80°C. Cell pellets were lysed in a buffer containing 1% sodium deoxycholate in 100 mmol/L 4-(2-hydroxyethyl)-1-piperazineethanesulfonic acid assisted by sonication (Bioruptor Pico, Diagenode). Protein extracts were cleaned and desalted using a magnetic bead–based SP3 protocol (44). Following tryptic digestion off the SP3 beads, peptide sample were acidified using 1% aqueous formic acid, adjusted to 2% acetonitrile, spiked with a synthetic peptide standard used for retention time alignment (iRT Standard), and kept at +4°C for further analysis.

Samples were analyzed on a nanoflow chromatography system (nanoRSLC, Thermo Fisher Scientific) hyphenated to a hybrid timed ion mobility–quadrupole–time-of-flight mass spectrometer (timsTOF Pro 2, Bruker) or a hybrid quadrupole–Orbitrap mass spectrometer (Exploris 480, Thermo Fisher Scientific). In brief, 400 ng (timsTOF Pro2) or 800 ng (Exploris 480) equivalents of peptides were enriched on a reversed-phase C18 trapping column (0.3 cm × 300 μm, Thermo Fisher Scientific) and separated on a reversed-phase C18 column with an integrated CaptiveSpray Emitter (Aurora 25 cm × 75 μm, IonOpticks) using a 100 minutes linear gradient of 5% to 34% acetonitrile/0.1% formic acid (v:v) at 200 nL min^−1^ and a column temperature of 50°C.

Data-independent acquisition (DIA) analysis on the timsTOF Pro 2 instrument was performed in diaPASEF mode (45) using a 20 × 2 variable size window acquisition method from *m/z* 400 to 1,200 to include the 2+/3+/4+ population in the *m/z*–ion mobility plane. The collision energy was ramped linearly as a function of the mobility from 59 eV at 1/K_0_ = 1.6 Vs cm^−2^ to 20 eV at 1/K_0_ = 0.6 Vs cm^−2^. DIA analysis on the Exploris 480 instrument was performed in DIA mode using 30 variable size window acquisition method from *m/z* 350 to 1,250 using a normalized collision energy setting of 25%, an MS1 resolution setting of 120,000 full width half maximum (FWHM), and an MS2 resolution setting of 30,000 FWHM. Two technical replicates per biological replicate were acquired.

Protein identification was achieved using the Pulsar algorithm in Spectronaut Software v19.1 (Biognosys) using default settings. All DIA data were searched against the UniProtKB *Homo sapiens* reference proteome (revision 09/2024) augmented with a set of 54 known common laboratory contaminants at default settings. For quantitation, up to the 6 most abundant fragment ion traces per peptide and up to the 10 most abundant peptides per protein were integrated and summed up to provide protein area values. Mass and retention time calibration as well as the corresponding extraction tolerances were dynamically determined. Both identification and quantification results were trimmed to a FDR of 1% using a forward-and-reverse decoy database strategy (46, 47).

Protein quantification values were logarithmically transformed and technical duplicates were averaged. All proteins were annotated by gene names. The combination treatment and control were statistically compared with limma package (v3.62.2) in R (v4.4.2). A preranked GSEA was conducted on the protein list sorted by log_2_ FC. GSEA software from the BROAD institute was used (v4.4.4; RRID: SCR_003199).

### Drug synergy analysis

The zero interaction potency (ZIP), Loewe additivity, and highest single agent (HSA) synergy scores without correction were determined using the SynergyFinder web application (RRID: SCR_019318) using default settings (synergyfinder.fimm.fi, v3.0; ref. 48), and the most synergistic area score is shown.

### Chorioallantoic membrane assay

Chorioallantoic membrane (CAM) assays were performed as previously described (49). Briefly, fertilized eggs (VALO Biomedia) were incubated for 3 days at 37.8°C and 60% humidity before windowing. Egg viability was confirmed on day 7. On the same day, PSN1 cells were seeded in T175 flasks. On day 8, cells were pretreated with the following drugs: indisulam (240 nmol/L), MRTX1719 (100 nmol/L), or a combination of indisulam (240 nmol/L) + MRTX1719 (100 nmol/L). After 2 days of pretreatment, cells were trypsinized, and 3 million cells per egg were mixed with 60 μL Matrigel (Th.Geyer GmbH) supplemented with the respective inhibitor at the concentrations mentioned above. The cell–Matrigel mixture was applied to the CAM on day 10, and eggs were incubated for an additional week. On day 17, tumors were harvested, washed with PBS, and photographed using a binocular microscope (Olympus Europe). Tumors were then transferred to 35% and 70% ethanol (1 hour each). For fixation and staining, tumors were incubated overnight at RT under slow rotation in 4% paraformaldehyde (Serva Electrophoresis GmBH) in PBS (#70011044, Thermo Fisher Scientific, pH 7.4, Invitrogen, pH 7.4) supplemented with 0.7% phosphotungstic acid (#HT152-250ML, Sigma-Aldrich) diluted in 70% ethanol. Samples were rinsed briefly in water and stored in fresh 70% ethanol. Tumors were subsequently dehydrated using ascending ethanol concentrations and embedded in paraffin (#10054 Suesse Labortechnik GmBH und Co. KG). Tumor volume was estimated using the prolate spheroid formula: V = $\frac{4}{3}{\pi a}^{2}c$, in which a = semi-axis of symmetry and c = polar semi-axis.

### Zebrafish maintenance and tumor growth assay

Wild-type zebrafish (*Danio rerio*) were obtained from the Zebrafish International Resource Center and maintained under standard conditions, as previously described (50), at 28.5°C in a core aquatic facility. For tumor xenograft assays, MiaPaCa-2 cells were labeled with calcein (1 μg/mL, #C1430, Thermo Fisher Scientific) and cultured with the drugs for 2 or 5 days prior to injection. Approximately 500 cells per embryo were injected above the duct of Cuvier into the perivitelline space of the embryo at 48 hours after fertilization, as previously described (51). Following injection, embryos were incubated in fish water containing MRTX1719 (30 nmol/L) and/or indisulam (300 nmol/L) or 600 nmol/L indisulam with or without 100 nmol/L MRTX1719 for 72 hours. DMSO served as the vehicle control. Tumor imaging was performed at 24, 48, and 72 hours after injection using a Nikon Eclipse TS100 microscope (Nikon). Tumor size and staining cell intensity were evaluated based on the area of the developed tumors. All animal experiments were approved by the Institutional Animal Care and Use Committee of the University of Bordeaux and conducted in accordance with the National Advisory Committee for Laboratory Animal Research Guidelines, under authorization from the French regulatory authorities.

### Statistics

Statistical analyses were performed using GraphPad Prism (RRID: SCR_002798; GraphPad Software). Data normality was assessed using the Shapiro–Wilk test, followed by the appropriate parametric or nonparametric tests as indicated. The statistical tests used for each analysis are specified in the figure legends. Multiple-testing correction was applied as described in the respective figure legends. Unless otherwise stated, all experiments were performed in at least three independent biological replicates. In all figures, data are presented as the mean ± standard deviation. ISRA outgrowth curves were plotted as Kaplan–Meier curves and compared using the log-rank test. *P* values are given as follows: *, *P* < 0.05; **, *P* < 0.01; ***, *P* < 0.001; ****, *P* < 0.0001.

### Ethics approval statement and patient consent statement

The primary human PDAC cellular models were established and analyzed in accordance with the Declaration of Helsinki and approved by the local ethical committee at University Medical Center Göttingen (vote May 11, 2017). The written informed consent from the patients for research use was obtained prior to the investigation.

## Results

### Induction of the splicing machinery upon PRMT inhibition

**Figure 1.**
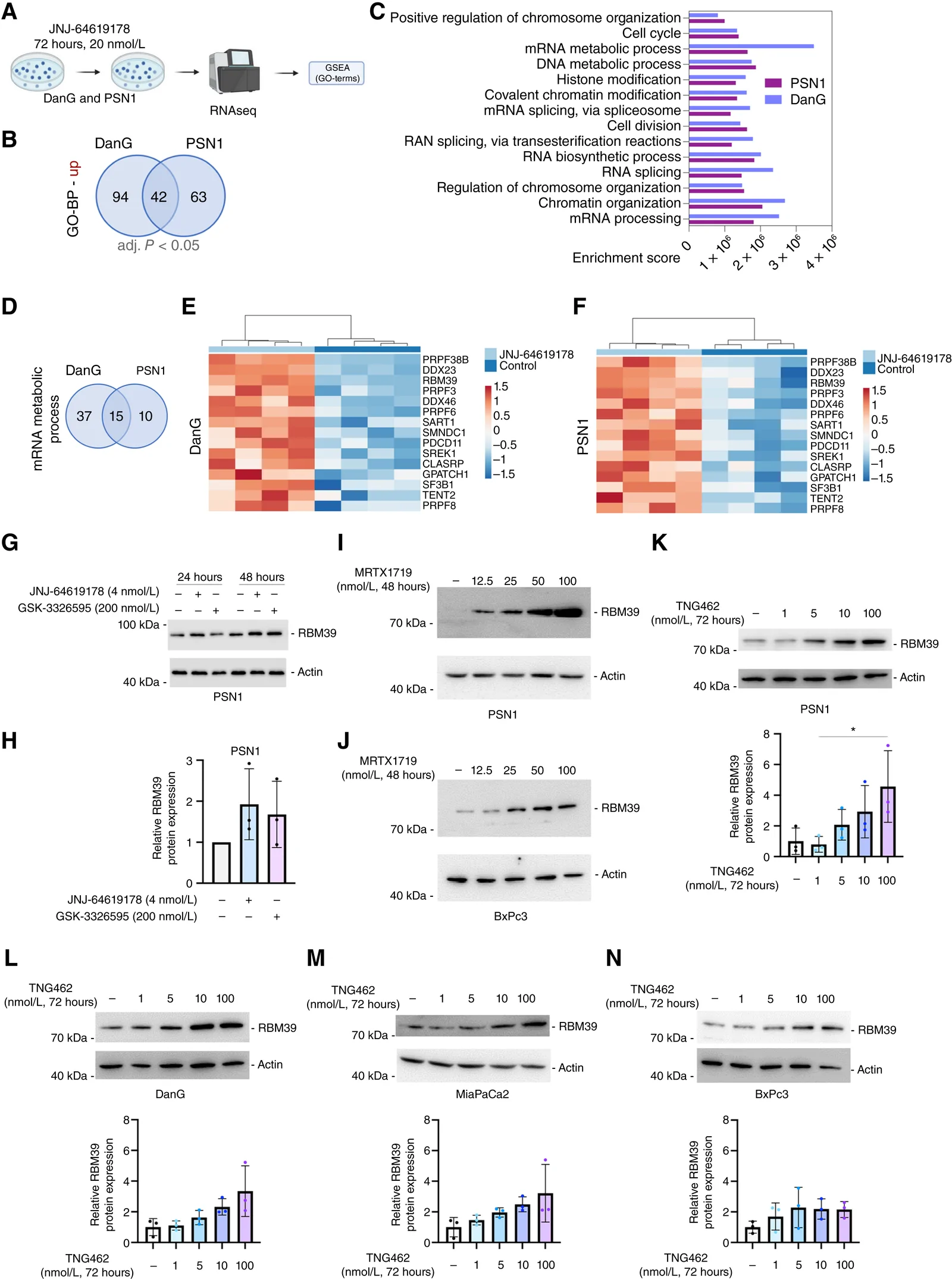
Adaptation to PRMT5 inhibition involves upregulation of splicing factors. **A,** Scheme of RNA-seq experiment: DanG and PSN1 cells were treated for 72 hours with 20 nmol/L JNJ-64619178 or the vehicle control. RNA-seq data were analyzed by GSEA. **B,** Venn diagram of upregulated GO-BP gene sets in JNJ-64619178–treated DanG and PSN1 cells. **C,** GSEA (GeneTrail 3.2) results of GO-BP signatures in JNJ-64619178–treated vs. DMSO control DanG and PSN1 cells. Depicted are the top 14 enriched gene sets sorted by the lowest mean adjusted *P* value. Enrichment scores are shown on the *x*-axis. **D,** Venn diagram of JNJ-64619178 upregulated genes with a gene score >4 (GeneTrail 3.2) in the GO-BP mRNA metabolic process gene set. **E** and **F,** Heatmap of the 15 genes (**D**) upregulated in DanG (**E**) and PSN1 (**F**) cells by JNJ-64619178 treatment. **G,** Western Blot of RBM39 expression in PSN1 cells treated with PRMT5i JNJ-64619178 (4 nmol/L) or GSK-3326595 (200 nmol/L) or DMSO as the vehicle control for 24 and 48 hours. β-Actin was used as the loading control. One of three representative biological replicates is shown. **H,** Quantification of RBM39 expression (**G**) after 48 hours (*n* = 3). The intensity of antibody signal of RBM39 was normalized to the loading control. **I** and **J,** Western Blot of RBM39 protein expression in PSN1 (**I**) and BxPc3 (**J**) cells treated with increasing dosages of MRTX1719 for 48 hours. β-Actin was used as the loading control, and one of two (PSN1) or three (BxPc3) biological replicates is shown. **K–N,** Top, Western blot of RBM39 protein expression in PSN1 (**K**), DanG (**L**), MiaPaCa2 (**M**), and BxPc3 (**N**) cells treated with increasing dosages of TNG462 for 72 hours. β-Actin was used as the loading control. Bottom, quantification of three independent experiments. **K,** Friedman test *P* = 0.0009, *Dunn multiple-comparison test: *P* < 0.05; (**L**) Friedman test *P* = 0.015; (**M**) Friedman test *P* = 0.026; (**N**) Friedman test *P* = 0.038. [**A,** Created in BioRender. Schneider, G. (2026) https://BioRender.com/8or8uh9.]

We recently identified a PRMT5i-sensitive subtype of PDAC linked to the MYC oncogene (4). Given that cellular adaptation to PRMT5is is incompletely understood, we investigated transcriptome data from the PDAC cell lines PSN1 and DanG (4), treated for 72 hours with 20 nmol/L of the PRMT5i JNJ-64619178 (Fig. 1A). Using GO-BP signatures, we identified 42 signatures commonly upregulated by the PRMT5i JNJ-64619178 (Fig. 1B). Prioritizing by the lowest mean adjusted *P* value (top 14 signatures), we found a marked enrichment of mRNA splicing–related signatures among the top hits (Fig. 1C). Additionally, signatures associated with chromatin biology and cell division were also induced by PRMT5i treatment (Fig. 1C). To define genes connected to mRNA homeostasis and upregulated in both PDAC cell lines, we used the gene score (>4) from the GO-BP mRNA metabolic process signature determined in GeneTrail3 (34), which identified 15 commonly upregulated genes (Fig. 1D–F). Due to its druggability, we focused on RBM39, a member of the U2AF-like family of RNA-binding proteins with described functions in pre-mRNA splicing (52–54). *RBM39* mRNA was found to be induced by PRMT5 inhibition in both cell lines (Fig. 1E and F). In addition to JNJ-64619178, we included the PRMT5i GSK-3326595. Both PRMT5is induced RBM39 protein expression in PSN1 cells after 48 hours of treatment (Fig. 1G and H), even at the low concentrations of 4 nmol/L JNJ-64619178 used in the experiment. In addition, we utilized published data from the MTA-cooperative PRMT5i AMG193 and the experimental lead compound AM-9747 (data accessible via NCBI GEO: GSE273376; ref. 14). Twenty-nine percent of PDAC cell lines harbor a deletion of *MTAP* (Supplementary Fig. S1A; ref. 14). Consistent with our previous observations (4), AMG193-sensitive cells were characterized by enrichment of MYC- and splicing-related signatures (Supplementary Fig. S1B). Additionally, an increased expression of the *RBM39* mRNA was detected in BxPc3 and MiaPaCa2 cells upon a 6-day treatment period with AM-9747 (Supplementary Fig. S1C). In the investigated dataset, the upregulation of *RBM39* mRNA by AM-9747 was marginal in PSN1 cells (Supplementary Fig. S1C). Furthermore, splicing signatures were induced by AM-9747 in two of three investigated PDAC lines (Supplementary Fig. S1D and S1E). To further support our findings, we used an additional MTA-cooperative PRMT5i, MTRX1719 (12) and TNG462 (15). MRTX1719 dose-dependently increased the expression of RBM39 48 hours after the treatment in PSN1 (Fig. 1I; Supplementary Fig. S1F) and BxPc3 cells (Fig. 1J; Supplementary Fig. S1G). No u-regulation of RBM39 protein expression upon 48 hours of treatment with the indicated doses of MRTX1719 was observed in DanG cells (Supplementary Fig. S1H). In addition, RBM39 protein expression was upregulated in PDAC cells 72 hours following treatment with TNG462 (Fig. 1K–N). We therefore conclude that adaptation to PRMT5 inhibition is associated with increased expression of genes controlling mRNA homeostasis.

**Figure 2.**
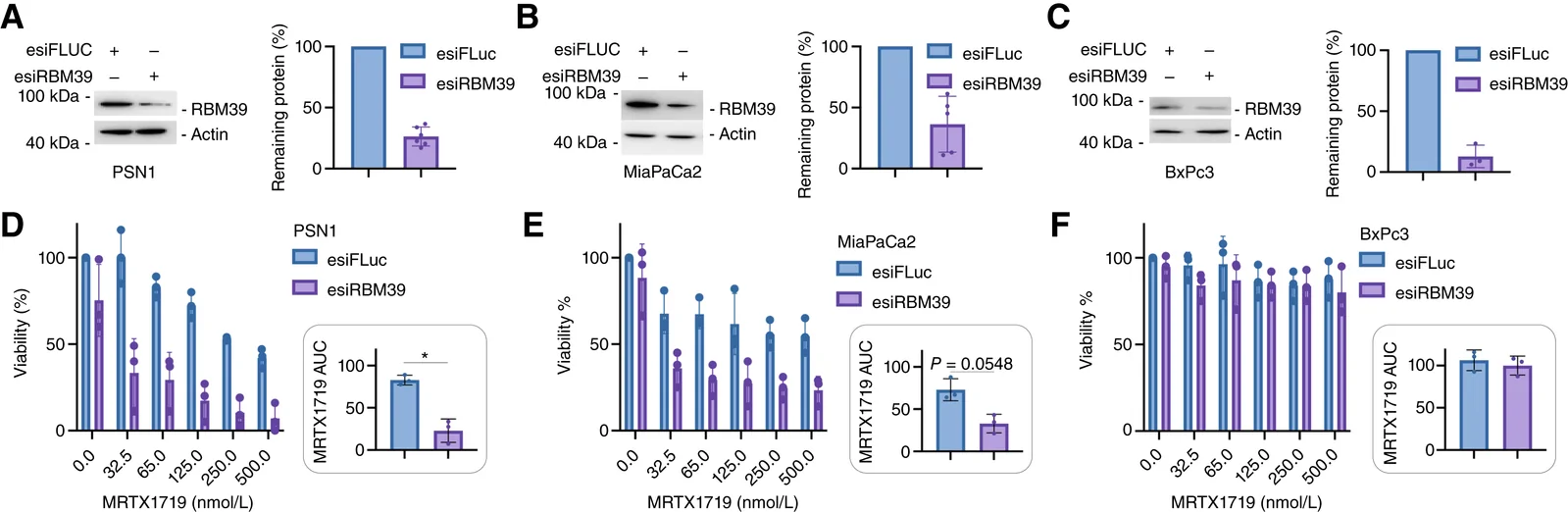
RBM39 knockdown increased MRTX1719 responsiveness. **A–C,** PSN1 (**A**), MiaPaCa2 (**B**), and BxPc3 (**C**) cells were transfected with the indicated esiRNAs. Left, Western blot of RBM39 expression 72 hours after the transfection. β-Actin was used as the loading control. Right, quantification of RBM39 expression of (**A**) *n* = 6, (**B**) *n* = 5, and (**C**) *n* = 3 biological replicates. **D–F,** PSN1 (**D**), MiaPaCa2 (**E**), and BxPc3 (**F**) were transfected with the indicated siRNAs and treated after 24 hours with different dosages of MRTX1719 as indicated for additional 72 hours. Left, viability normalized to cells treated with the vehicle control and esiFLUC-transfected cells is depicted. Right, areas under the dose–response curves (AUC) were calculated and compared. Statistical significance was assessed using a two-tailed paired *t* test; *, *P* < 0.05 or the exact *P* value is indicated.

Because PRMT5 inhibition increased the expression of RBM39, we used siRNA targeting RBM39 to test its functional relevance (Fig. 2A–C). In both MiaPaCa2 and PSN1 cells, RBM39 knockdown enhanced MRTX1719 response (Fig. 2D and E). BxPc3 cells shows limited responsiveness to MRTX1719, and RBM39 knockdown confers only a marginal additional effect (Fig. 2F), indicating a context-specific response.

Together, our data show that PRMT5 inhibition in PDAC cells induces genes linked to mRNA splicing and homeostasis, notably upregulating RBM39. Functional experiments revealed that RBM39 knockdown enhances PRMT5i efficacy in some PDAC models, highlighting its role in adaptive resistance.

### Indisulam-sensitive PDACs show enrichment of splicing signatures

**Figure 3.**
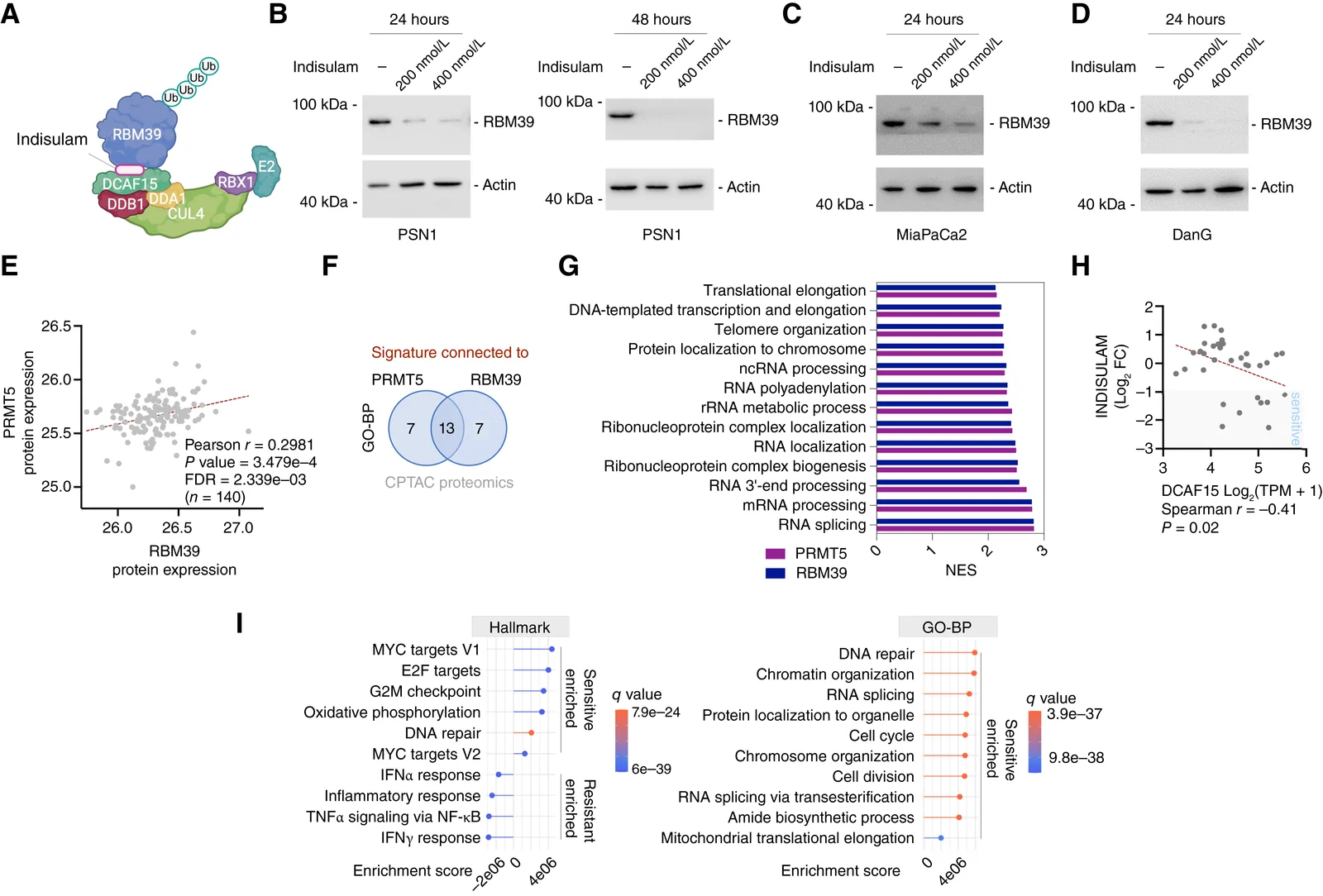
Activity of indisulam in PDAC models. **A,** Scheme of the mechanism of indisulam-dependent degradation of RBM39. **B,** Western blot of RBM39 expression of PSN1 cells treated with 0, 200, and 400 nmol/L indisulam for 24 (left) and 48 (right) hours. β-Actin was used as the loading control. One of three biological replicates is shown. **C** and **D,** Western blot of RBM39 expression of MiaPaCa2 (**C**) and DanG (**D**) cells treated with 0, 200, and 400 nmol/L indisulam for 24 hours. β-Actin was used as the loading control. One of three biological replicates is shown. **E,** Correlation analysis of PRMT5 (*y*-axis) and RBM39 (*x*-axis) protein expression using CPTAC PDAC proteomics data. For statistical analysis, the Pearson correlation coefficient, *P* value, and FDR are indicated. **F,** Venn diagram of the top 20 GO-BP gene sets connected to RBM39 and/or PRMT5 protein expression. Gene sets were determined via LinkInterpreter of LinkedOmics. **G,** Bar graph showing the commonly enriched GO-BP gene sets linked to RBM39 and PRMT5 determined in **F**. The normalized enrichment score (NES) is shown on the *x*-axis. **H,** Correlation analysis of indisulam sensitivity and DCAF15 expression in pancreatic cancer cell lines using DepMap data. Spearman’s *r* = −0.41, *P* = 0.02. **I,** GSEA (GeneTrail3.2) of Hallmark (left) and GO-BP (right) in indisulam-sensitive PDAC cell lines. The enrichment score is shown on the *x*-axis, and the *q* values are color-coded. [**A,** Created in BioRender. Schneider, G. (2026) https://BioRender.com/rzri1up.]

RBM39 can be selectively degraded by aryl sulfonamides such as indisulam which act as molecular glue that promote its interaction with the DCAF15-E3 ubiquitin ligase (Fig. 3A–D; Supplementary Fig. S2A–S2C; refs. 52, 53). Using the CPTAC PDAC proteomics data (36), we detected a positive correlation between PRMT5 and RBM39 protein expression (Fig. 3E). Furthermore, we observed common pathways connected to both proteins, with dominance in mRNA metabolic processes, including splicing (Fig. 3F and G). No correlation was detected between *PRMT5* and *RBM39* mRNA expression in three PDAC expression datasets (Supplementary Fig. S2D–S2F). In addition, the indisulam sensitivity scores of PDAC cell lines showed a modest negative correlation with *DCAF15* mRNA expression (Fig. 3H), and cell lines that are sensitive to indisulam are characterized by the enrichment of DNA repair, MYC activation, proproliferative, and splicing-related signatures (Fig. 3I). Therefore, our findings support an association between RBM39 and PRMT5 in PDAC.

### RBM39 degradation synergizes with PRMT5 inhibition in cell culture models

**Figure 4.**
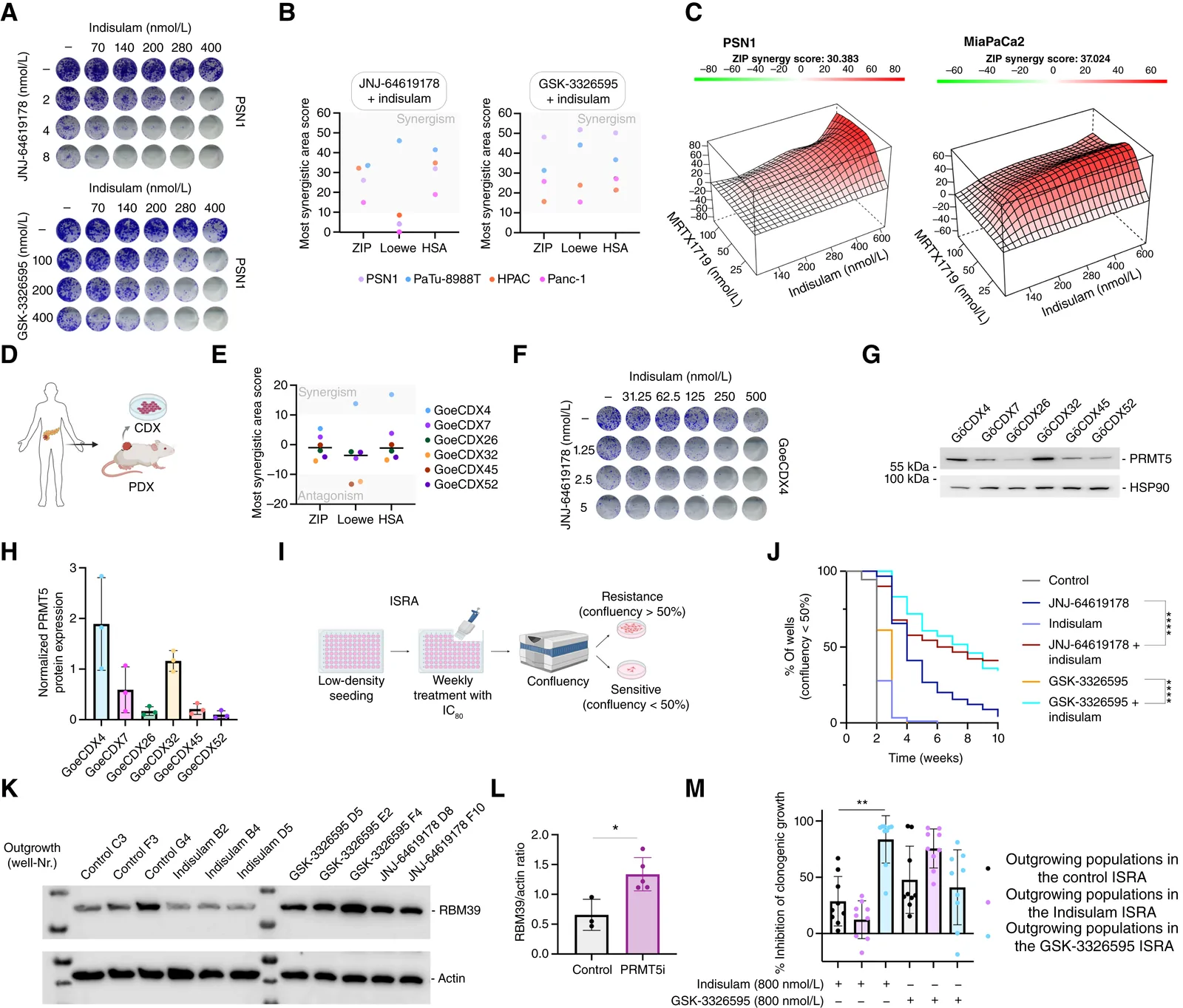
Synergistic response to PRMT5 and RBM39 inhibitors. **A,** Clonogenic assays of PSN1 cells treated with the indicated dosages of indisulam and JNJ-64619178 (above) or GSK-3326595 (below) for 9 days. One of three biological replicates is shown. **B,** Most synergistic area scores according to the ZIP, Loewe, and HSA models for the combination of indisulam with PRMT5 inhibition [JNJ-64619178 (left) or GSK-3326595 (right)] in four PDAC cell lines: PSN1, PaTu-8988T, HPAC, and Panc-1. Response values are from the corresponding clonogenic growth assays (*n* = 3) as determined in (**A**), and concentrations were adapted to the responsiveness of each cell line. **C,** Synergy distribution plots showing the ZIP synergy score of PSN1 (left) and MiaPaCa2 (right) cells treated with MRTX1719 and indisulam as indicated. Values are based on clonogenic growth assays (*n* = 3). **D,** Scheme of the generation of PDCLs. PDX, patient-derived xenotransplant. **E,** ZIP, Loewe, and HAS most synergistic area scores of six GoeCDX cell lines treated with JNJ-64619178 and indisulam. The experiment corresponds to at least three independent biological replicates for every cell line. **F,** Clonogenic assay of GoeCDX4 cells with the indicated dosages of indisulam and JNJ-64619178. One of six biological replicates is shown. **G,** Western blot of PRMT5 expression in GoeCDX cells. HSP90 was used as the loading control. One of three biological replicates is shown. **H,** Quantification of **G**. **I,** Scheme of the experimental setup of the ISRA. PSN1 cells were seeded at a low density and treated with the individual GI_80_ value of the indicated drugs and drug combinations (8 nmol/L JNJ-64619178, 800 nmol/L indisulam, 800 nmol/L GSK-3326595, 200 nmol/L GSK-3326595, and 400 nmol/L indisulam or 4 nmol/L JNJ-64619178 and 70 nmol/L indisulam; DMSO was used as the control). The medium with fresh therapies was changed weekly, and confluence was measured weekly for 10 weeks. Wells with a confluence more than 50% were considered resistant. **J,** Results of the ISRA plotted as a Kaplan–Meier curve Statistical analysis was performed with a log-rank test: ****, *P* < 0.0001. The results from three biological replicates (3 × 30 wells per condition) are shown. **K,** ISRA outgrown cell populations from the indicated conditions were further cultured in medium containing the respective drug. RBM39 protein levels were assessed by Western blotting. β-Actin served as a loading control. **L,** Quantification of the RBM39/β-actin ratio in control (*n* = 3 outgrown wells) and PRMT5i (*n* = 3 GSK3326595 and *n* = 2 JNJ-64619178 outgrown wells). Mann–Whitney test: *, *P* < 0.05. **M,** Cell populations that grew out in the control ISRA, GSK-3326595 ISRA, and indisulam ISRA were treated with vehicle (DMSO), 800 nmol/L indisulam, or 800 nmol/L GSK-3326595. Relative clonogenic growth was assessed after 7 days. The bar graph shows the percentage inhibition of clonogenic growth normalized to DMSO-treated controls, and each dot represents one population derived from a single well. For each biological replicate of the ISRA (**J**), three populations were analyzed, yielding a total of *n* = 9 population tested in one biological replicate. Statistical analysis was performed using the Kruskal–Wallis test with Dunn multiple-comparison test: **, *P* < 0.01. [**D,** Created in BioRender. Schneider, G. (2026) https://BioRender.com/0w6dd14; **I,** Created in BioRender. Schneider, G. (2026) https://BioRender.com/xsbxjh0.]

To translate our findings into a combinatorial therapy concept, we tested the PRMT5i JNJ-64619178 and GSK-3326595 in combination with indisulam and determined the ZIP, Loewe additivity, and HSA scores. Overall, the data support a synergistic interaction in PSN1, PaTu-8988T, HPAC, and Panc-1 cells (Fig. 4A and B). Furthermore, the MTA-cooperative PRMT5i MRTX1719 showed synergistic activity with indisulam in the *MTAP*-deleted PDAC cell lines PSN1 and MiaPaCa2 (Fig. 4C), whereas TNG462 also synergized with indisulam in DanG and PSN1 cells (Supplementary Fig. S2G–S2I). Additionally, we observed a synergistic effect of JNJ-64619178 and indisulam in one of six tested primary PDCLs (Fig. 4D–F). This line exhibited the highest PRMT5 protein expression among the PDCLs examined (Fig. 4G and H). Variable RBM39 expression was detected across these PDCLs, and GoeCDX4, GoeCDX7, and GoeCDX26 were negative for MTAP protein expression (Supplementary Fig. S2J). To explore resistance development and assess the superiority of the PRMT5i and indisulam combination, we conducted ISRA in PSN1 cells (Fig. 4I; ref. 26). We applied GI_80_ doses of the monotherapies and combination therapy to PSN1 cells. The therapeutic effects of GSK-3326595 and indisulam were modest (Fig. 4J). Whereas JNJ-64619178 significantly delayed outgrowth, the assay demonstrated the superior efficacy of combining GSK-3326595 or JNJ-64619178 with indisulam (Fig. 4J). To understand adaptive responses to PRMT5 inhibition, we expanded cells from three randomly selected outgrown wells per condition of each replicate of the ISRA in medium containing the drug at its GI_80_ concentration. Notably, the cell populations that emerged from PRMT5i -treated wells showed elevated RBM39 protein expression (Fig. 4K and L). Comparative analysis of indisulam sensitivity revealed enhanced drug response in outgrown populations adapted to GSK-3326595 (Fig. 4M). These findings suggest that PRMT5 blockade induces RBM39 upregulation and may consequently increase dependency on RBM39. We evaluated the effects of indisulam and MRTX1719 in a MiaPaCa2 zebrafish xenograft model and a PSN1 chorioallantoic membrane model but observed no synergistic interaction between the two drugs (Supplementary Fig. S3A–S3C). The combination of PRMT5 inhibition with indisulam showed synergy in selected PDAC cell lines and patient-derived models, but the effect was not observed the investigated *in vivo* models.

### Characterization of the indisulam and PRMT5i response

**Figure 5.**
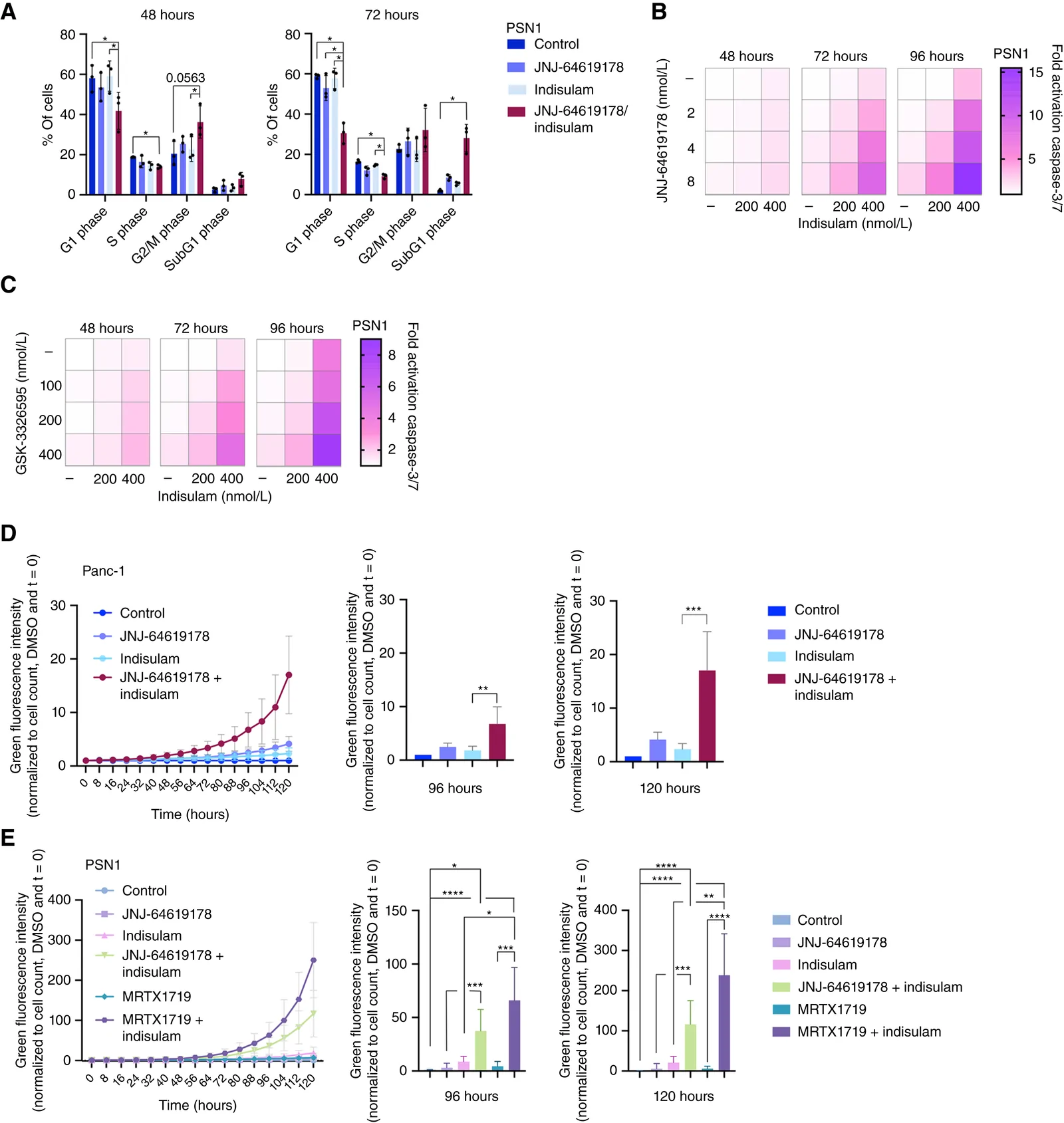
Combined PRMT5 and RBM39 inhibition induced apoptosis. **A,** Cell-cycle distribution of PSN1 cells treated with the vehicle control (DMSO), 4 nmol/L JNJ-64619178, 200 nmol/L indisulam, or the combination of 4 nmol/L JNJ-64619178 and 200 nmol/L indisulam for 48 (left) and 72 hours (right). Cell-cycle distribution was determined by FACS of PI-stained cells (*n* = 3). Repeated measure one-way ANOVA with correction for multiple-testing according to the Tukey test. The *P* value is indicated or *, *P* < 0.05. **B** and **C,** Caspase-3/7 Assay of PSN1 cells treated with the indicated dosages of indisulam and JNJ-64619178 (**B**) or GSK-3326595 (**C**) for 48, 72, and 96 hours. The fold activation of caspase-3/7 is color-coded (*n* = 3). **D,** Panc-1 caspase reporter cells were treated with 8 nmol/L JNJ-64619178 and/or 400 nmol/L indisulam, or DMSO was used as the vehicle control. The green fluorescence intensity (caspase activation) normalized to cell count, DMSO control, and t = 0 is shown over time (left) at 96 hours (middle) and 120 hours (right). Ninety-six hours: Friedman test, *P* < 0.0001; 120 hours: Friedman test, *P* < 0.0001. The *P* value after Dunn multiple-comparison test is indicated: **, *P* < 0.01; ***, *P* < 0.001, *n* = 3. **E,** PSN1 caspase reporter cells were treated with PRMT5i (8 nmol/L JNJ-64619178 or 100 nmol/L MRTX1719) and/or 400 nmol/L indisulam. DMSO was used as the vehicle control. The green fluorescence intensity (caspase activation) normalized to cell count, DMSO control, and t = 0 is shown over time (left) at 96 hours (middle) and 120 hours (right). Ninety-six hours: Friedman test, *P* < 0.0001; 120 hours: Friedman test, *P* < 0.0001. Dunn multiple-comparison test: *, *P* < 0.05; **, *P* < 0.01; ***, *P* < 0.001; ****, *P* < 0.0001, *n* = 3.

PRMT5 inhibition induces retention of detained introns (DI), particularly in transcripts encoding cell-cycle regulators such as the mitotic kinase Aurora B (AURKB; ref. 55) also in the PDAC context (56). Furthermore, our recent work demonstrates that PRMT5 inhibition leads to significant downregulation of AURKB in the context of PDAC, an effect connected to an arrest of the cell cycle in the G2/M phase of the cell cycle (4). At the used doses, the combination of JNJ-64619178 and indisulam demonstrated superior efficacy compared with either agent alone, as evidenced by an increase of cells in G2/M phase accompanied by corresponding reductions in both G1- and S-phase cell populations (Fig. 5A). Similar cell-cycle profiles were induced by MRTX1719 and indisulam (Supplementary Fig. S4). Given the observed increase in sub-G1 phase cells following JNJ-64619178 and indisulam combination treatment, we quantified caspase-3/7 activity to assess apoptosis induction. Over the treatment course, both JNJ-64619178 and GSK-3326595 demonstrated time-dependent induction of caspase-3/7 activity when combined with indisulam (Fig. 5B and C). To further validate and quantify apoptotic induction, we used a GFP-based fluorogenic split caspase reporter system (22) following recently established protocols (23). In Panc-1 cells, the JNJ-64619178 and indisulam combination significantly enhanced caspase activity compared with monotherapies (Fig. 5D). This synergistic effect was similarly observed in PSN1 cells, in which both JNJ-64619178 and MRTX1719 demonstrated superior caspase induction when combined with indisulam (Fig. 5E). Notably, the combinatorial effect manifested after 72 hours of treatment and progressively intensified over time (Fig. 5D and E). Collectively, these findings demonstrate that the combination treatment affects both cell-cycle progression and cell death.

Our published findings, corroborated by independent studies, identify the PRMT5–MYC axis as a relevant regulator of PDAC aggressiveness (4, 57). Deregulated MYC in PDAC creates targetable vulnerabilities (58–62), including PRMT5 inhibition (4). To assess a potential role of MYC in the indisulam response, we used MYC-CRISPRa–activated HPAC cells (Supplementary Fig. S5A; ref. 4). This model activates MYC expression driven from the endogenous promoter and is characterized by activation of the MYC program, including the upregulation of spliceosomal components (Supplementary Fig. S5B). Consistent with MYC’s established role in regulating the splicing machinery (63), MYC-CRISPRa–activated HPAC cells exhibited enhanced sensitivity to indisulam (Supplementary Fig. S5C), suggesting the functional interplay between MYC-driven splicing dysregulation and vulnerability to RBM39 degradation. Notably, PRMT5i treatment induced RBM39 upregulation in MYC-CRISPRa–activated HPAC cells as well as in the empty vector control (Supplementary Fig. S5D). MYC-CRISPRa–activated HPAC cells exhibited enhanced sensitivity to the tested monotherapies and the combined PRMT5 inhibition and indisulam treatment (Supplementary Fig. S5E).

### Dual PRMT5 and RBM39 inhibition interferes with cell-cycle, DNA repair, and metabolic processes

**Figure 6.**
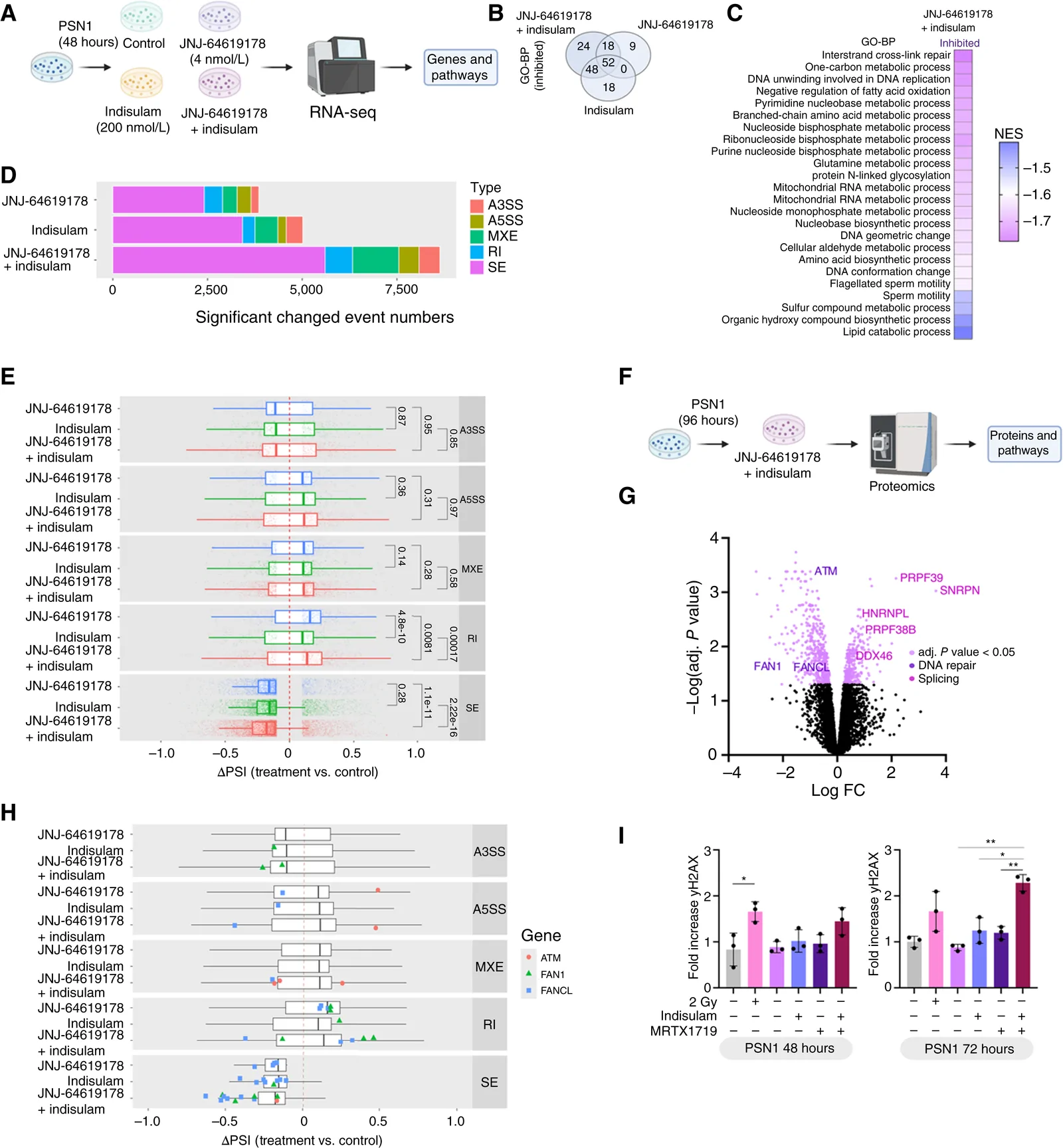
PRMT5 and RBM39 inhibition regulate DNA repair and metabolic pathways. **A,** Workflow of the RNA-seq approach: PSN1 cells were treated with DMSO as the vehicle control, 4 nmol/L JNJ-64619178, 200 nmol/L indisulam, or the combination of 4 nmol/L JNJ-64619178 and 200 nmol/L indisulam for 48 hours, mRNA was isolated, and mRNA sequencing with subsequent GSEA was performed. The experiment was conducted as four biological replicates. **B,** Venn diagram showing downregulated GO-BP gene sets in PSN1 cells; all treatments were compared with the vehicle control in individual GSEAs. Only gene sets with an FDR < 0.05 were considered significant and used for the analysis. **C,** 24 GO-BP gene sets downregulated exclusively in the combination treatment are indicated. The value of the normalized enrichment score (NES) is color-coded. **D,** Number and composition of significant changed alternative splicing (AS) events observed upon monotherapy or combination treatment of JNJ-64619178 and/or indisulam. **E,** Percent spliced-in (ΔPSI) distribution of different AS events identified following monotherapy or combination treatment with JNJ-64619178 and/or indisulam. *P* values based on the Wilcoxon test are indicated. **F,** PSN1 cells were either treated with DMSO control or 4 nmol/L JNJ-64619178 and 200 nmol/L indisulam for 96 hours followed by mass spectrometry determination of the proteome. **G,** Volcano plot of deregulated proteins in PSN1 cells treated with the combination therapy [see (**F**)] vs. control. Proteins with an adjusted *P* value < 0.05 were colored. Proteins involved in the DNA repair pathway are highlighted lilac, whereas proteins involved in the splicing pathway are highlighted pink. **H,** ΔPSI distribution of different AS events in the DNA damage signaling and repair pathway genes ATM, FAN1, and FANCL observed upon monotherapy or the combination treatment with JNJ-64619178 and/or indisulam. **I,** Results of the γH2A.X phosphorylation assay: PSN1 cells were treated with 50 nmol/L MRTX1719 and/or 240 nmol/L indisulam for 48 hours (left) or 72 hours (right). For negative control, cells were left vehicle-treated; for positive control, cells were irradiated with 2 Gy for 30 minutes. The fold increase of γH2A.X phosphorylation is shown on the *y*-axis. Statistical analysis was performed using a repeated-measures one-way ANOVA with correction for multiple-testing according to the Tukey test: **, *P* < 0.01; *, *P* < 0.05, *n* = 3. A3SS, alternative 3′ splice site; A5SS, alternative 5′ splice site; MSE, mutually exclusive exon; RI, retained intron; SI, skipped exon. [**A,** Created in BioRender. Schneider, G. (2026) https://BioRender.com/fwl2ir3; **F,** Created in BioRender. Schneider, G. (2026) https://BioRender.com/z84e701.]

To elucidate the mechanistic basis of the observed synergism, we conducted transcriptomic profiling in PSN1 cells (Fig. 6A; Supplementary Table S1). Consistent with our previous findings, JNJ-64619178 treatment significantly enriched spliceosome-related gene signatures (Supplementary Fig. S6A) and *RBM39* mRNA expression (Supplementary Fig. S6B), further validating the critical role of splicing modulation in the PRMT5i-induced response. Indisulam treatment also elevated RBM39 mRNA expression (Supplementary Fig. S6B), suggesting the activation of a compensatory regulatory mechanism. Pathway analysis of the different treatment groups revealed selective depletion of 24 GO-BP signatures specifically in PSN1 cells treated with the combination therapy (Fig. 6B). These significantly depleted signatures were functionally associated with metabolic processes, DNA repair, and DNA replication pathways (Fig. 6C). Furthermore, we observed a distinct increase in alternative splicing events, particularly in cells in which PRMT5 and RBM39 were coinhibited (Fig. 6D). Whereas inhibition of PRMT5 or RBM39 affected all classes of differential splicing events, the combination therapy preferentially affected exon skipping (Fig. 6D and E). Integration of splicing events with Hallmark pathway analysis highlighted a prominence of cell-cycle regulation and DNA repair pathways (Supplementary Fig. S6C).

To extend these findings at the protein level, we performed quantitative mass spectrometry analysis (Fig. 6F; Supplementary Table S2). Notably, proteomic analysis revealed significant downregulation of key DNA repair and DNA damage signaling proteins, including ATM, FAN1, and FANCL (Fig. 6G). Furthermore, we observed treatment-induced splicing events across all three genes (Fig. 6H). Deregulated splicing of numerous DNA damage response and repair genes was recently demonstrated in breast cancer stem cells following PRMT5 inhibition (64).

Nucleotides play key roles in essential cellular processes such as DNA replication, repair, transcription, and ribosome biogenesis (65), all of which are frequently hyperactive in cancer cells. Given the observed inactivation of nucleotide metabolism signatures and downregulation of DNA repair/damage signaling proteins, we assessed DNA damage markers. Seventy-two hours after the MRTX1719 and indisulam treatment, we detected increased H2AX phosphorylation (Fig. 6I). We next analyzed differentially expressed proteins following JNJ-64619178 and indisulam treatment using GSEA. Consistent with the mRNA data, metabolic processes were suppressed under the combination therapy. Notably, signatures linked to amino acid transport and metabolism were prominently downregulated (Supplementary Fig. S6D). In addition, we observed significant enrichment of immune-related signaling pathways in cells treated with the PRMT5i and indisulam combination (Supplementary Fig. S6D). Strikingly, despite dual targeting of the splicing machinery with PRMT5i and indisulam, splicing signatures (Supplementary Fig. S6D) and proteins (Fig. 6G) were markedly upregulated, a finding consistent with cellular adaptation mechanisms observed for PRMT5i monotherapy. The protein-level findings are mirrored in the transcriptome profiles, in which the combination therapy inhibited MYC and metabolic signatures while activating inflammatory pathways (Supplementary Fig. S6E). Together, these findings demonstrate that coinhibition of PRMT5 and RBM39 modulates the splicing landscape, alters cancer-relevant pathways, and induces of immune- and inflammation-related gene signatures.

## Discussion

Despite significant progress in other cancer entities, PDAC remains the most lethal solid malignancy, with a persistently dismal 5-year survival rate of only 13%. Current standard-of-care therapies, which predominantly consist of moderately effective chemotherapy combinations, offer limited clinical benefit (66, 67). PRMT5 is a promising therapeutic target in PDAC, with MTA-cooperative inhibitors exploiting synthetic lethality in the ∼20% to 30% of cancers harboring *CDKN2A–MTAP* codeletion (2, 68). This notion is further supported by second-line data reported for vopimetostat (TNG462) in PDAC (reported by the company; refs. 15, 21). Given the prevalence of primary and secondary resistances also to MTA-cooperative PRMT5is (16, 17), combinatorial strategies are needed to enhance and sustain responses to PRMT5 inhibition. Consistent with this rationale, PRMT5is show therapeutic synergy with KRAS inhibitors (2, 56, 69), a strategy already under clinical evaluation in PDAC (e.g., NCT06333951 and NCT06922591).

To identify rational PRMT5i combinations, we systematically analyzed therapy-induced adaptive responses. Our studies reveal that PRMT5 inhibition triggers an adaptive program in PDAC, characterized by consistent upregulation of splicing-related genes. These findings were conserved across different PRMT5i and PDAC model systems. The role of PRMT5 in splicing is one of its best-understood functions (70). The PRMT5–MEP50 complex, in conjunction with the cofactor CLNS1A, catalyzes sDMA of the spliceosomal Sm proteins D1, D3, and B/B′ (71–74). These arginine-methylated Sm proteins with higher binding affinity for survival of motor neuron assemble into a heptameric ring that binds small-nuclear RNA, forming the core components of the spliceosome (70, 75). The connection between PRMT5 and splicing is functionally relevant for PRMT5-associated phenotypes. In glioblastoma, PRMT5 inhibition induces a specific splicing alteration involving DIs (55). mRNAs containing DIs are retained in the nucleus and consequently not translated (76, 77). Furthermore, recent studies have revealed that the majority of splicing alterations caused by PRMT5 inhibition involve DIs, which exhibit a high degree of conservation across diverse cancer cell lines (78). Pathways enriched among genes containing DIs overlap with known PRMT5 functions, including proliferation, DNA repair and damage response, replication, and splicing regulation (78), consistent with the splicing changes observed in our analysis. Careful comparison of PRMT5 inhibition with CLNS1A degradation in cancer cells established that methylosome-dependent regulation of Sm protein methylation and splicing fidelity is a key contributor to the cellular response (78). Consistently, the interaction of PRMT5 with CLNS1A was demonstrated to be necessary for Sm protein methylation and genetic depletion of CLNS1A impaired growth of *MTAP*-deleted PDAC cell lines (79). We interpret these findings as consistent with our observations that PRMT5 inhibition induces upregulation of splicing regulators, pointing to a compensatory adaptation mechanism. However, additional PRMT5-controlled processes may contribute to the observed phenotypes.

To disrupt adaptation to PRMT5 inhibition, we chose the aryl sulfonamide indisulam, which acts as a molecular glue to promote DCAF15-E3 ubiquitin ligase–mediated degradation of the SR-rich protein RBM39 (52, 53). RBM39 functions as both a transcriptional cofactor, for example for the estrogen receptor, AP-1, and NF-κB, and as a splicing factor within the U2AF-like protein family, controlling exon skipping and intron retention (54). Moreover, indisulam has been evaluated in multiple clinical trials in which it showed tolerable safety profiles, pointing to some translational potential of our findings (54). In our experiments, we demonstrated the upregulation of RBM39 in PDAC cells upon short- and long-term PRMT5i treatment. Blocking this response with indisulam was associated with increased cell death and DNA damage as evidenced by H2A.X phosphorylation and down-egulation of proteins involved in DNA damage repair.

Our work opens several avenues for future investigation. Although RBM39’s role in mRNA splicing is established (54), we cannot exclude additional functions—such as acting as a transcriptional cofactor—that may contribute to the observed synergy between indisulam and PRMT5 inhibition. RBM39 regulates hepatocellular carcinoma cell metabolism by directly binding and sensing arginine, coupling arginine uptake to transcriptional reprogramming of cancer metabolism (80). Although our transcriptomic and proteomic analyses indeed indicate that combined PRMT5i and indisulam treatment regulates metabolic pathways—including amino acid and nucleoside metabolism—the specific role of arginine in these processes and the adaptation to PRMT5i treatment remain to be elucidated.

### Limitations of our study

The demonstration that the splicing machinery is a collateral vulnerability of MYC-driven cancers (63, 81) and can be targeted with RBM39 degradation (82) or PRMT5 inhibition (4, 83) is documented. Although the increased sensitivity to the combination of indisulam and PRMT5 inhibition observed in our MYC gain-of-function model suggest a connection, we have not mechanistically established this association.

The induction of RBM39 upon PRMT5 inhibition seems to be context-dependent, with the extent of regulation varying across PDAC models. Moreover, although RBM39 knockdown increased responsiveness to PRMT5 inhibition, we cannot exclude the possibility that other indisulam neosubstrates (84) also contribute to the observed synergy between PRMT5is and indisulam. It remains to be systematically clarified whether the upregulation of RBM39 is required for the described effects or whether the observed synergy can be explained solely by a combined disruption of the splicing machinery, requiring no RBM39 upregulation. In addition, the determinants of synergy between indisulam and PRMT5 inhibition remain to be clarified, particularly because only one of the investigated PDCLs showed a synergistic response to treatment with a first-generation PRMT5i and indisulam.

Our zebrafish and chorioallantoic membrane models demonstrated no overt enhancement in efficacy for the combination therapy compared with monotherapy. However, the cause of the discrepancy in the *in vivo* and *in vitro* models—whether due to dose scheduling, delayed cellular responses to the combination therapy, or injection of fragile pretreated cells—remains to be determined in future work. Furthermore, we currently cannot exclude the possibility that the *in vitro* and *in vivo* findings simply diverge. Interestingly, splicing modulators have been demonstrated to generate splicing-derived neoantigens that are presented on major histocompatibility complex class I molecules, thereby eliciting CD8^+^ T-cell responses (85). MRTX1719 enhances the susceptibility of *MTAP*-deleted tumors to T cell–mediated cytotoxicity, providing a mechanistic rationale for the improved efficacy of MRTX1719/anti–PD-1 combination therapy observed *in vivo* (86). Furthermore, in murine neuroblastoma models, indisulam-mediated RBM39 degradation creates an inflammatory microenvironment that enhances the antitumor activity of natural killer cells (87). Our omics profiling revealed activation of immune response and inflammatory signatures. Whereas these findings suggest potential immunologic benefits of the combination therapy, rigorous evaluation in immunocompetent models—though beyond the scope of this study—represents a possible avenue for future investigation.

### Conclusion

Our study identifies an adaptive response in which PRMT5 inhibition induces upregulation of components of the splicing machinery, including RBM39. Dual targeting of the splicing machinery has also been reported as a relevant combinatorial approach in acute myeloid leukemia models (88) or recently in *MYCN*-driven neuroblastoma (89). The synergy between RBM39 targeting and PRMT5 inhibition observed in selected cellular models suggests that the upregulation of the splicing machinery may contribute to cell survival. Although the long-term outgrowth was significantly delayed by dual splicing targeting, it might indicate a degree of redundancy within this system, thereby highlighting the importance of identifying and defining essential nodes in the adaptive program.

## Supplementary Material

Figure S1 Figures S1

Figure S2 Figure S2

Figure S3 Figure S3

Figure S4 Figure S4

Figure S5 Figure S5

Figure S6 Figure S6

Table S1 Table S1

Table S2 Table S2

## Data Availability

mRNA expression datasets of PRMT5i, indisulam, and combinatorial treated PDAC cells can be accessed via ENA: PRJEB95000. Proteomics profiles of control and PRMT5i- and RBM39-treated PSN1 data can be accessed via Proteomics IDEntifications Database (PRIDE): PXD073473. Other data generated in this study are available upon request to the corresponding author.
