# Supplementary material for: Targeting PRMT5 Inhibitor–Induced Adaptation in Pancreatic Cancer with the RBM39 Degrader Indisulam
Source: Cancer Res Commun. 2026 Sep 1;6(9):2039–55. doi: 10.1158/2767-9764.CRC-25-0670 (PMC13530115; doi:10.1158/2767-9764.CRC-25-0670)
Supplement: Supplementary file 1 [file crc-25-0670_figure_s1_suppsf1.pdf]

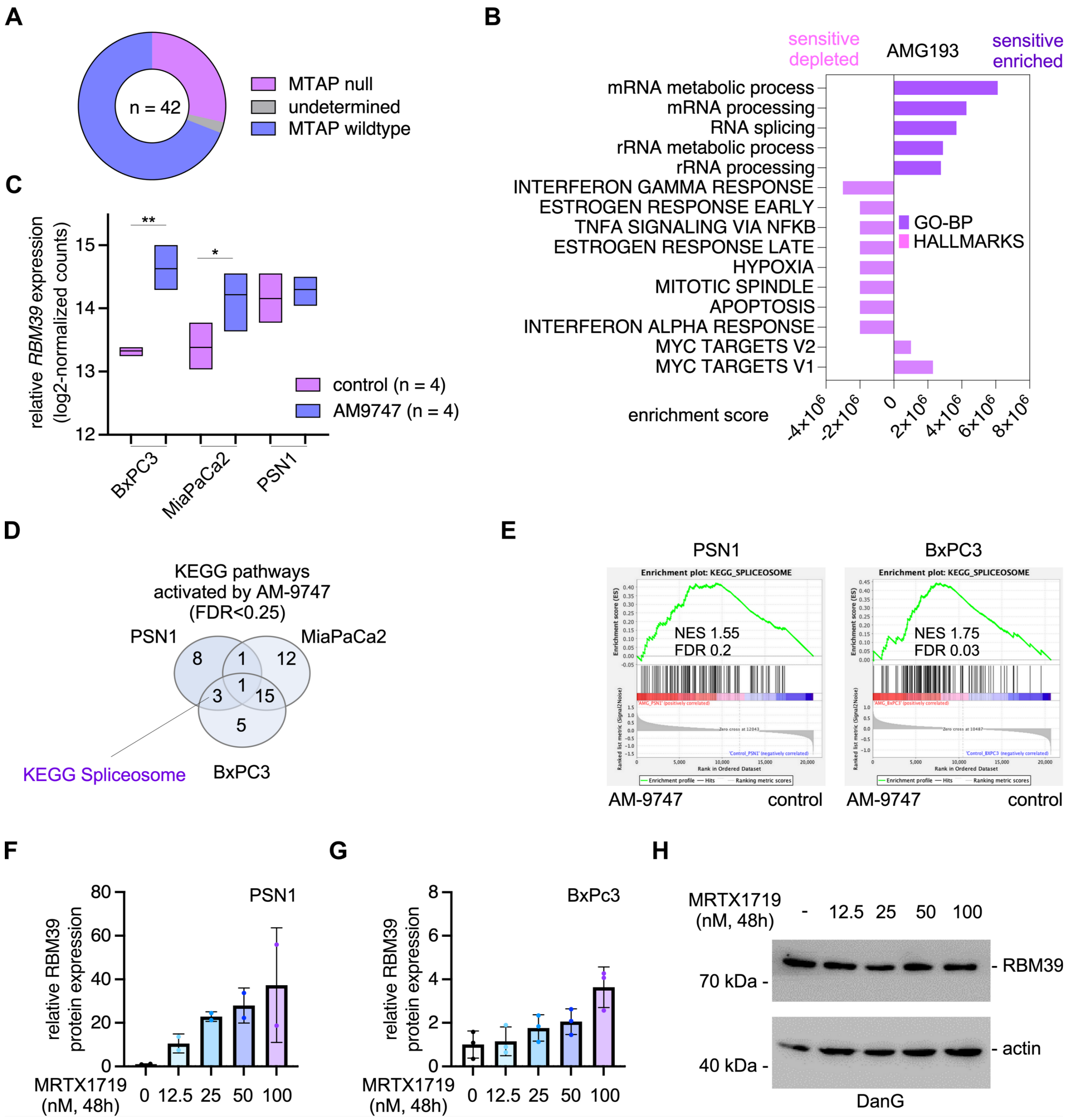

Figure S1: Activation of splicing signatures upon PRMT5 treatment.

(A) Pie chart of the MTAP status of 42 PDAC cell lines, 29% of PDAC cell lines showed deletion of MTAP. Data are accessible via NCBI GEO: GSE273376. **(B)** GSEA (GeneTrail3.2) of AMG193 sensitive PDAC cell lines using GO-BP (lilac) and HALLMARK (pink) signatures. The enrichment score is shown on the x-axis. **(C)** RBM39 mRNA expression of BxPC3, MiaPaca2 and PSN1 cells treated for six days with AMG-9794 or vehicle control. On the y-axis the relative RBM39 expression (log2, normalized) is given and the adjusted p value: ** $p < 0.01$, * $p < 0.05$. **(D)** Venn Diagram of KEGG pathways (GSEA App 4.4) upregulated by treatment with the

PRMT5i AM-9747 in PSN1, BxPC3 and MiaPaCa2 cells. Only gene sets with a false discovery rate (FDR) <0.25 were considered as significant. The Spliceosome pathway is enriched in PSN1 and BxPc3. **(E)** Enrichment plots of KEGG Spliceosome pathway in PSN1 (left) and BxPc3 (right) corresponding to (D). **(F, G)** Western blots of RBM39 protein expression in PSN1 (F) (n = 2) and BxPC3 (G) (n = 3) cells treated with increasing dosages of MRTX1719 for 48 hours were quantified and normalized to the loading control. F: Friedman test p = 0.12; G: Friedman test p = 0.12. **(H)** Western Blot of RBM39 protein expression in DanG cells treated with increasing dosages of MRTX1719 for 48 hours. β-Actin was used as loading control and one of two biological replicates is shown.
