# Supplementary material for: Targeting PRMT5 Inhibitor–Induced Adaptation in Pancreatic Cancer with the RBM39 Degrader Indisulam
Source: Cancer Res Commun. 2026 Sep 1;6(9):2039–55. doi: 10.1158/2767-9764.CRC-25-0670 (PMC13530115; doi:10.1158/2767-9764.CRC-25-0670)
Supplement: Supplementary file 2 [file crc-25-0670_figure_s2_suppsf2.pdf]

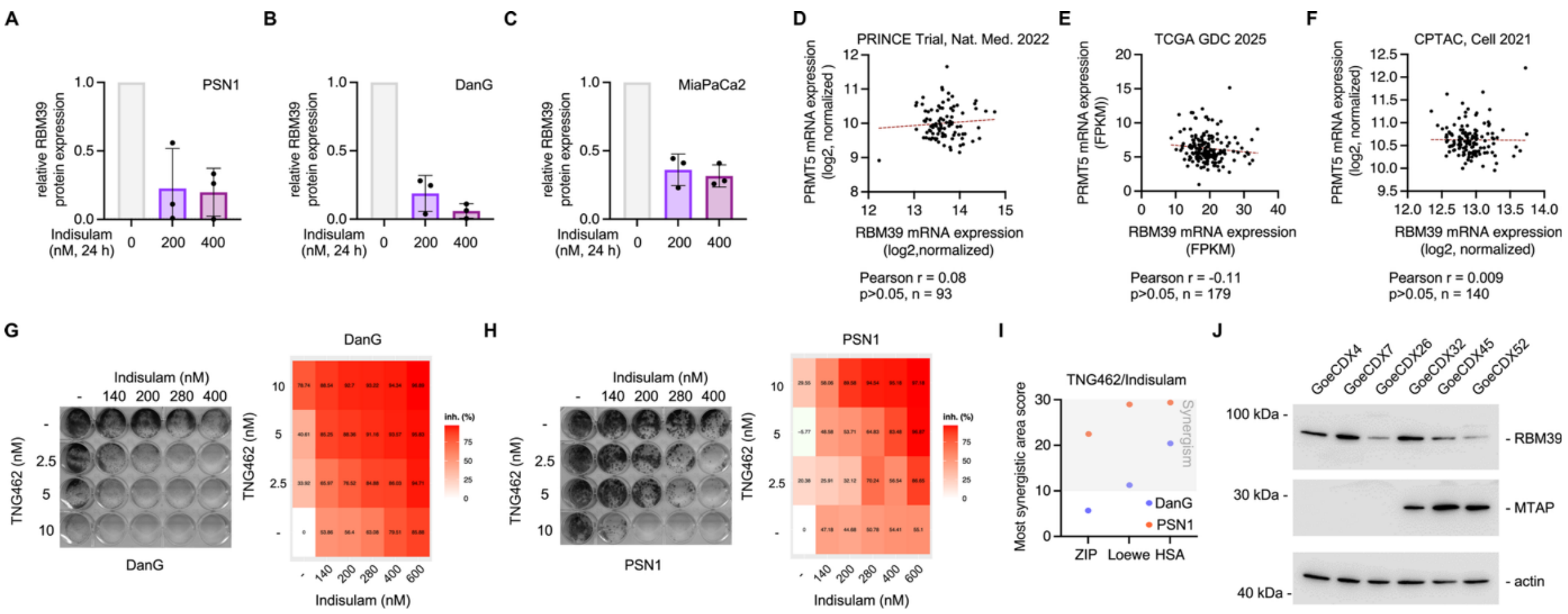

**Figure S2:** Indisulam and PRMT5 association.

**(A-C)** Quantification of western blots of RBM39 expression of PSN1 (A), DanG (B), and MiaPaCa2 (C) cells treated with 0, 200 and 400 nM Indisulam for 24 hours. The RBM39-to-actin ratio in untreated cells was set to 1, and the relative RBM39 expression is shown. **(D-F)** The mRNA expression levels of PRMT5 and RBM39 were correlated in the indicated datasets, and the Pearson correlation coefficient is shown. **(G-H)** *Left panels*: Clonogenic assays of DanG (G) and PSN1 (H) cells treated with the indicated concentrations of Indisulam and TNG462. One of three biological replicates is shown. *Right panel:* Percentage inhibition matrices for the indicated dose combinations. **(I)** Most synergistic area scores according to the Zero Interaction Potency (ZIP), Loewe, and Highest Single Agent (HSA) models for the combination of Indisulam with TNG462 in DanG and PSN1 cells. Response values are from the corresponding clonogenic growth assays (n = 3) as determined in (G-H). **(J)** Western Blot of RBM39 and MTAP expression in GoeCDX cells. β-actin was used as loading control. One of three biological replicates is shown.
