# Supplementary material for: Targeting PRMT5 Inhibitor–Induced Adaptation in Pancreatic Cancer with the RBM39 Degrader Indisulam
Source: Cancer Res Commun. 2026 Sep 1;6(9):2039–55. doi: 10.1158/2767-9764.CRC-25-0670 (PMC13530115; doi:10.1158/2767-9764.CRC-25-0670)
Supplement: Supplementary file 3 [file crc-25-0670_figure_s3_suppsf3.pdf]

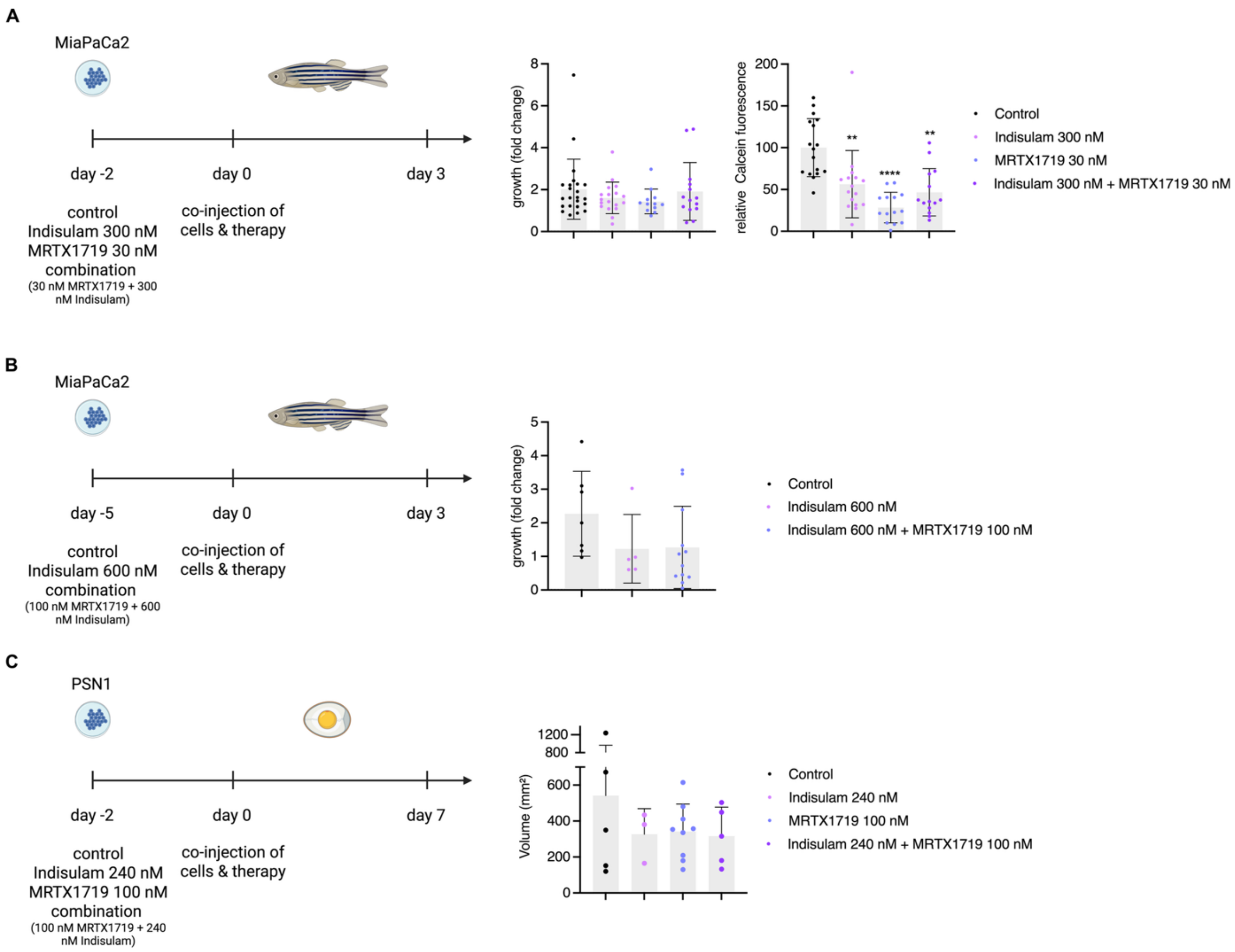

Figure S3: PRMT5 and RBM39 inhibition in Zebrafish Xenografts and CAM PDAC models. **(A)** Left: MiaPaca2 cells were treated 2 days with the indicated drugs (DMSO control, 30 nM MRTX1719 and/ or 300 nM Indisulam) prior to injection in the duct of Cuvier in the zebrafish embryo. The embryos were incubated in fish water containing the drugs. Pictures were taken on day 0 and 2 and the tumor area was calculated. Middle: Tumor growth (depicted as fold change on the y-axis), the tumors were treated as indicated. Every dot represents a tumor; error bars show the SD. Right: For every tumor, the relative Calcein fluorescence was determined, every dot represents a fluorescence measurement in one fish. Kruskal-Wallis test $p < 0.0001$, Dunn`s multiple comparison test: **** $p < 0.0001$, ** $p < 0.01$. **(B)** Left: MiaPaca2 cells were treated 5 days with the indicated drugs (DMSO control, 100 nM MRTX1719 and/or 600 nM Indisulam) prior to injection in the duct of Cuvier in the zebrafish embryo. Pictures were taken on day 0 and 2 and the tumor surface area was calculated. Right: Tumor growth (depicted as fold change on the y-axis), the tumors were treated as indicated. Every dot represents a tumor; error bars show the SD. (A) and (B) Created in BioRender. Schneider, G.

(2026) https://BioRender.com/g18jcyb. Only (A) is depicted in https://BioRender.com/g18jcyb. **(C)** Left: Treatment scheme of the CAM assay: PSN1 cells were treated with DMSO as vehicle control, 100 nM MRTX1719, 240 nM Indisulam and the combination of MRTX1719 and Indisulam. 2 days later, the pretreated cells + the therapy were given onto the chorioallantoic membrane of a 10-day old chicken embryo. The tumor growth was assessed after 7 days. Right: Results of the CAM assay measured by tumor volume (depicted on the y-axis), every tumor is indicated as a dot, the error bars show the SD. Created in BioRender. Schneider, G. (2026) https://BioRender.com/wuzxq8m.
