# Supplementary material for: Targeting PRMT5 Inhibitor–Induced Adaptation in Pancreatic Cancer with the RBM39 Degrader Indisulam
Source: Cancer Res Commun. 2026 Sep 1;6(9):2039–55. doi: 10.1158/2767-9764.CRC-25-0670 (PMC13530115; doi:10.1158/2767-9764.CRC-25-0670)
Supplement: Supplementary file 4 [file crc-25-0670_figure_s4_suppsf4.pdf]

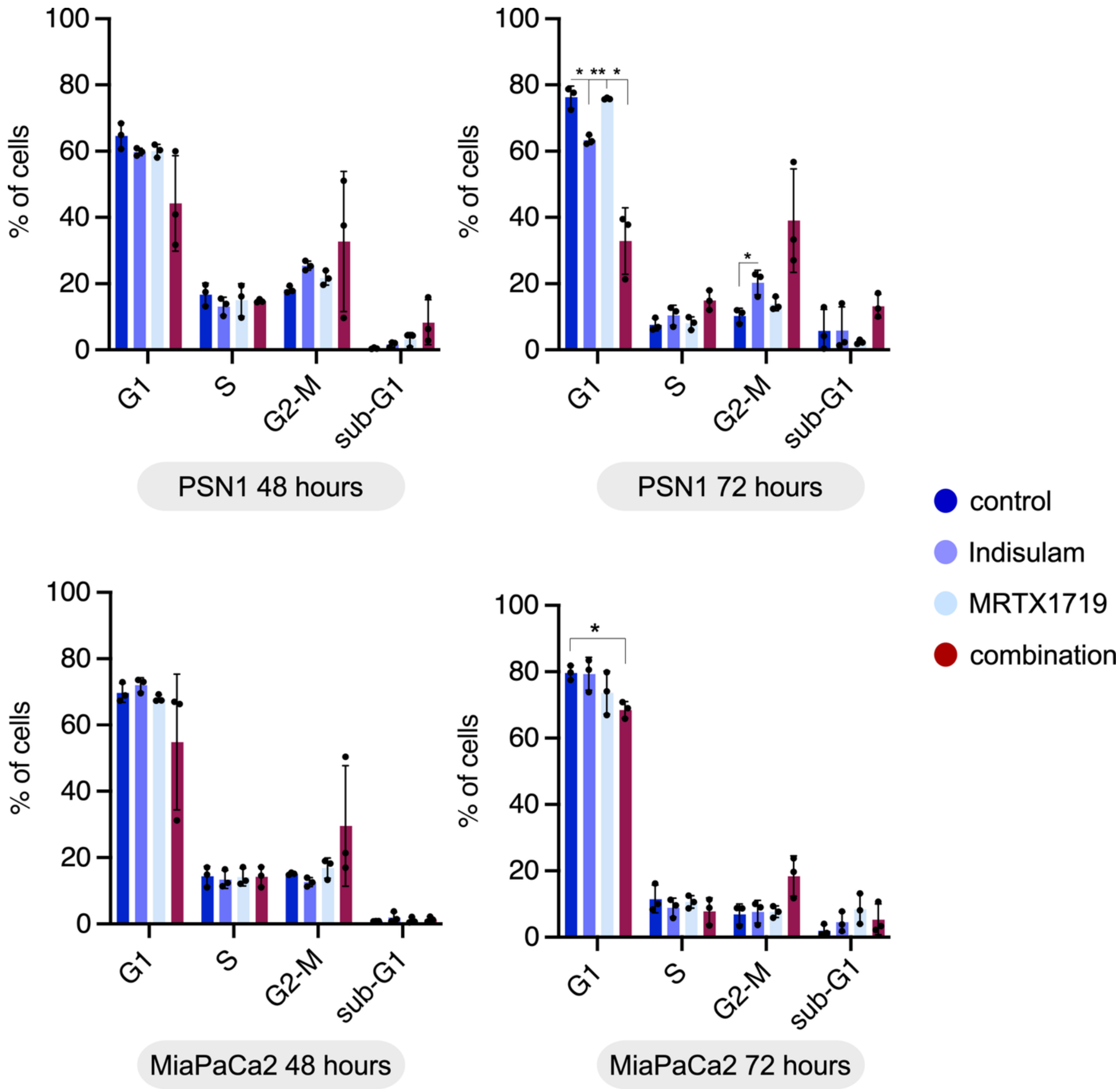

Figure S4: Cell cycle alteration upon PRMT5 inhibition and Indisulam treatment.

Cell cycle distribution of the indicated cell lines (above PSN1 and below MiaPaCa2). Cell cycle distribution was determined by FACS of propidium iodide–stained cells. Cells were treated with 50 nM MRTX1719, 240 nM Indisulam, the combination of MRTX1719 and Indisulam or DMSO for 48 (left) and 72 (right) hours. Results of 3 biological replicates were shown. Repeated-measures ANOVA: * $p < 0.05$, ** $p < 0.01$.
