# Supplementary material for: Targeting PRMT5 Inhibitor–Induced Adaptation in Pancreatic Cancer with the RBM39 Degrader Indisulam
Source: Cancer Res Commun. 2026 Sep 1;6(9):2039–55. doi: 10.1158/2767-9764.CRC-25-0670 (PMC13530115; doi:10.1158/2767-9764.CRC-25-0670)
Supplement: Supplementary file 5 [file crc-25-0670_figure_s5_suppsf5.pdf]

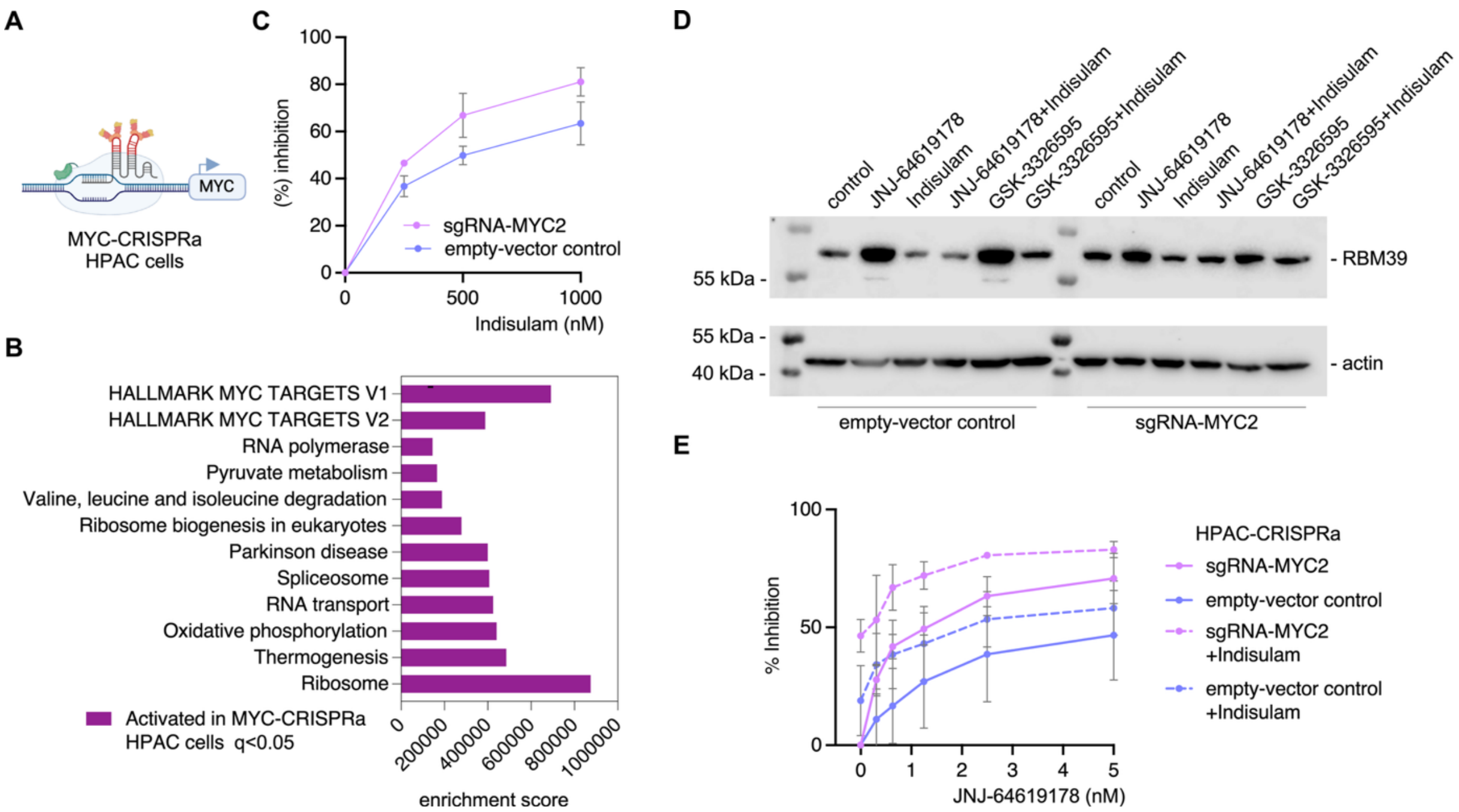

Figure S5: Indisulam and PRMT5i responsiveness of a MYC gain-of-function HPAC model. **(A)** MYC is activated in HPAC using CRISPRa synergistic activation mediator system (SAM). Created in BioRender. Schneider, G. (2026) https://BioRender.com/i55t125. **(B)** GSEA analysis of mRNA sequencing data from activated MYC-CRISPRa HPAC cells compared to the empty vector control. Only gene sets with a q-value <0.05 were counted as significant and are shown. The enrichment score is shown on the x-axis. **(C)** HPAC control (empty vector control) and MYC-CRISPRa HPAC cells were treated with Indisulam for long-term (clonogenic growth), the inhibition (y-axis) in dependency of the Indisulam concentration (x-axis) is shown (n = 3). **(D)** HPAC control (empty vector control) and MYC-CRISPRa (sgRNA-MYC2) HPAC cells were treated for 48 hours with a PRMT5i (5 nM JNJ-64619178, or 500 nM GSK-3326595) and/or 500 nM Indisulam or DMSO as vehicle control. Western Blot showing the RBM39 protein expression in response to indicated treatments. β-Actin was used as loading control. One of three biological replicates is shown. **(E)** HPAC control (empty vector control) and MYC-CRISPRa (sgRNA-MYC2) HPAC cells were treated with increasing dosages of JNJ-64619178 with and without 100 nM Indisulam. The inhibition rate (normalized to vehicle control (DMSO) treated cells, y-axis) in dependency of the JNJ-64619178 concentration (x-axis) is shown. The mean with SD of n = 3 biological replicates is depicted.
