# Supplementary material for: Targeting PRMT5 Inhibitor–Induced Adaptation in Pancreatic Cancer with the RBM39 Degrader Indisulam
Source: Cancer Res Commun. 2026 Sep 1;6(9):2039–55. doi: 10.1158/2767-9764.CRC-25-0670 (PMC13530115; doi:10.1158/2767-9764.CRC-25-0670)
Supplement: Supplementary file 6 [file crc-25-0670_figure_s6_suppsf6.pdf]

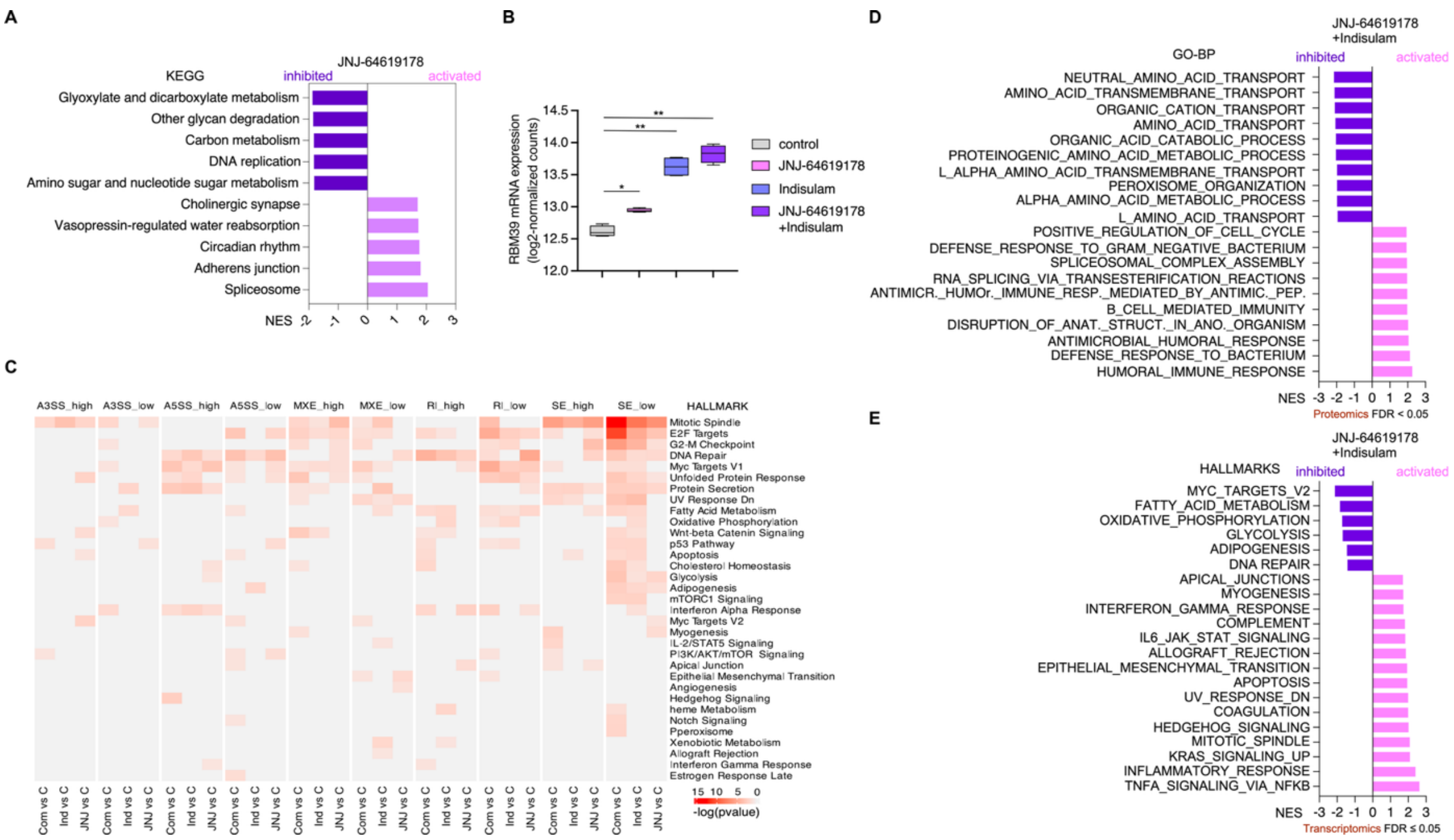

Figure S6: Pathways altered by combined PRMT5 and RBM39 inhibition.

(A) GSEA using KEGG gene sets in PSN1 cells treated with 4 nM JNJ-64619178 for 48 hours vs. DMSO control. The top 5 enriched and depleted gene sets based on the normalized enrichment score (NES, shown on x-axis) are shown. Only gene sets with a FDR < 0.05 are shown. **(B)** mRNA expression of RBM39 in PSN1 cells treated as indicated in Fig. 6A

shown as a box and whisker plot with whiskers from minimum to maximum, the line indicates the mean value. For statistical analysis repeated-measures ANOVA with correction according to Tukey`s was used: ** p < 0.01, * p < 0.05. **(C)** Hallmark pathway enrichment of genes with the most increased and decreased different AS events (ΔPSI ≤ -0.1 or ≥ 0.1) following mono- or combination therapy treatment of JNJ-64619178 and Indisulam compared to DMSO control. The p value is color coded. **(D)** Results of GSEA showing up- and downregulated gene sets based on protein expression in PSN1 cells treated with the combination vs. DMSO control. The logFC was used as a ranked in the pre-ranked GSEA. The NES is shown on the x-axis, only gene sets with an FDR <0.05 are shown. **(E)** Results of GSEA based on the transcriptomics analysis. HALLMARK signatures of PSN1 cells treated with the combination (JNJ-64619178 and Indisulam) vs. DMSO control are depicted. The NES is shown on the x-axis, only gene sets with an FDR <0.05 are shown.
